# Long-Term Efficacy and Safety of RNAi-Mediated Virus Resistance in ‘HoneySweet’ Plum

**DOI:** 10.3389/fpls.2021.726881

**Published:** 2021-10-12

**Authors:** Khushwant Singh, Ann M. Callahan, Brenda J. Smith, Tadeusz Malinowski, Ralph Scorza, Jana Jarošová, Eva Beoni, Jaroslav Polák, Jiban Kumar Kundu, Chris Dardick

**Affiliations:** ^1^Innovative Fruit Production, Improvement and Protection, Appalachian Fruit Research Station, Agricultural Research Service (USDA), Kearneysville, WV, United States; ^2^Division of Crop Protection and Plant Health, Crop Research Institute, Prague, Czech Republic; ^3^Department of Nutritional Sciences, Oklahoma State University, Stillwater, OK, United States; ^4^The National Research Institute of Horticulture, Skierniewice, Poland

**Keywords:** transgene, feeding studies, GM, small RNAs, RNA expression

## Abstract

Interfering RNA technology has been established as an effective strategy to protect plants against viral infection. Despite this success, interfering RNA (RNAi) has rarely been applied due to the regulatory barriers that confront genetically engineered plants and concerns over possible environmental and health risks posed by non-endogenous small RNAs. ‘HoneySweet’ was developed as a virus-resistant plum variety that is protected by an RNAi-mediated process against Sharka disease caused by the plum pox virus. ‘HoneySweet’ has been approved for cultivation in the United States but not in countries where the plum pox virus is endemic. In this study, we evaluated the long-term efficacy of virus resistance in ‘HoneySweet,’ the nature and stability of its sRNA profile, and the potential health risks of consuming ‘HoneySweet’ plums. Graft-challenged ‘HoneySweet’ trees carrying large non-transgenic infected limbs remained virus-free after more than 10 years in the field, and the viral sequences from the non-transgenic infected limbs showed no evidence of adaptation to the RNAi-based resistance. Small RNA profiling revealed that transgene-derived sRNA levels were stable across different environments and, on average, were more than 10 times lower than those present in symptom-less fruits from virus-infected trees. Comprehensive 90-day mouse feeding studies showed no adverse health impacts in mice, and there was no evidence for potential siRNA off-target pathologies predicted by comparisons of the most abundant transgene-derived sRNAs to the mouse genome. Collectively, the data confirmed that RNAi provides a highly effective, stable, and safe strategy to combat virus diseases in crop plants.

## Introduction

The recent decision by the European Food Safety Authority (EFSA) to regulate gene-edited crops has sparked a re-evaluation of past and future advanced breeding strategies for crop improvement. Strategies to develop virus-resistant crop plants helped to bring about the dawn of genetic engineering. Virus-resistant papaya and squash were among the first commercialized products derived from genetic engineering in the mid-1990s (Gottula and Fuchs, 2009). These first-generation crops were developed based on the concept of pathogen-derived resistance (PDR), which relied on the overexpression of viral coat protein genes in the plant (Beachy, 1997). It was not until the late 1990s that RNA interference (RNAi) mechanisms were discovered and later attributed to the resistance observed in some of these plants (Fire et al., 1998; Ruiz et al., 1998; Hamilton and Baulcombe, 1999). In recent years, new RNAi-based crops have been commercialized, including non-browning potatoes and apples, soybeans with improved oil content, alfalfa with reduced lignin, and low-acrylamide frying potatoes (Mat Jalaluddin et al., 2018). In addition, a slate of insect-resistant crops based on RNAi are now entering the markets (Zhang et al., 2017). This broader use of RNAi technology has prompted renewed concerns over its stability, long-term efficacy, and health and environmental risks.

The possible health effects caused by the consumption of small RNAs (sRNA) have been raised by the finding that insects are affected when consuming either plants producing RNAi targeted to the insect or consuming plants with RNAi applied ectopically (Zhang et al., 2017). For example, a modified bacterium living in the guts of bees produced an RNAi that killed the *Varroa* mites that consumed bee fat containing this RNAi (Leonard et al., 2020). These reports in insects demonstrate that it is possible to obtain RNAi-targeted effects through consumption, at least in some insects. A few publications have indicated that plant-derived sRNAs can downregulate genes in other animals and humans if sufficient homology exists and that these effects can lead to potential adverse health impacts (Zhang L. et al., 2012; Liu et al., 2017). Other researchers have refuted these findings as spurious (Dickinson et al., 2013; Tosar et al., 2014; Petrick et al., 2015; Chan and Snow, 2017). Studies such as these have fueled concerns that more complete information is necessary to confirm the safety of an RNAi approach (Heinemann et al., 2013).

Sharka is a serious disease of stone fruits that includes peaches, plums, apricots, and cherries. The disease, caused by the plum pox potyvirus (PPV), is marked by characteristic chlorotic rings on the leaves and fruits, premature fruit drop, and, in some cases, tree death. This disease was first recorded in Bulgaria in the early twentieth century and, since then, has spread throughout Europe and, more recently, to Asia, Africa, North, and South America, causing billions of dollars in losses to stone fruit industries worldwide (García et al., 2014). Natural resistance to the virus is very limited, with few reports of economically useful levels of resistance in the stone fruit germplasm (Vilanova et al., 2003; Hartmann and Neumüller, 2006; Zuriaga et al., 2013). Currently, PPV control efforts rely on intense monitoring, the destruction of infected trees, and the distribution of virus-free propagation materials, but these efforts have been insufficient to fully curb the spread of the disease (Rimbaud et al., 2015).

To combat PPV, the ‘HoneySweet’ plum (*Prunus domestica* L.) was developed in the early 1990s through a publicly funded US-French collaborative effort (Scorza et al., 1994). The original construct was designed to overexpress the PPV coat protein (CP) modeled after the papaya ringspot strategy that proved to be successful and is still in commercial use today (Gonsalves, 1998). Among more than 100 transgenic lines obtained, only a single PPV-resistant line (initially called C5) was identified and has since been released under the name ‘HoneySweet’ (Supplementary Figure 1) (Scorza et al., 2016). It was later discovered that recombination during the T-DNA insertion process resulted in a hairpin arrangement of the viral CP gene leading to RNAi-mediated virus resistance (Scorza et al., 2001). Over the past two decades, ‘HoneySweet’ trees have been evaluated in permitted field tests in the US, Spain, Poland, Romania, and the Czech Republic. ‘HoneySweet’ has proven to be a highly productive tree with excellent fruit quality and has been cleared for cultivation by the United States Department of Agriculture (USDA), the Environmental Protection Agency (EPA), and the Food and Drug Administration (FDA). International efforts are currently underway to obtain Canadian and European Union approval for ‘HoneySweet’ import and cultivation (Scorza et al., 2016). To address the possible risks of sRNAs in RNAi crops, we tested the long-term efficacy of ‘HoneySweet’ resistance in field-grown ‘HoneySweet’ trees and used sRNA expression profiling on trees grown in different environments to assess the composition and stability of transgene sRNAs relative to virus-infected trees. We then used that data to predict the potential sRNA off-target effects in mice as part of comprehensive 90-day feeding trials to screen for potential toxicological effects.

## Materials and Methods

### Germplasm and Locations

Graft-challenged trees: The plant material was collected from an experimental orchard of ‘HoneySweet’ grafted onto ‘St. Julian’ rootstock and planted in 2002 at a site in Praha, Czech Republic. The ‘HoneySweet’ grown in the Czech Republic were grown under the Ministry of Environment of the Czech Republic GM planting No. The 881/OER/GMO/01, and the field trial was extended with Ministry Reference Number 41538/ENV/09 issued on September 18, 2009. Eight of these trees were graft-inoculated in 2002 using PPV strain REC (PPV-REC)-infected buds of ‘Emma Leppermann.’ Leaves from three differently treated trees were evaluated: (A) one ‘HoneySweet’ tree with no virus-infected bud grafted on, (B) the eight ‘HoneySweet’ trees inoculated by PPV-REC using an infected graft of the cultivar ‘Emma Leppermann,’ which was removed from four of those trees after 5 years (in 2011), and C) infected ‘Emma Leppermann’ that had been grafted to the eight ‘HoneySweet’ trees (Supplementary Data sheet 2). Leaves were collected from individual trees in each treatment and were frozen at −80°C unless used immediately.

Multiple location trees: leaves and ripe fruits were collected from ‘HoneySweet’ in 2011 from two locations in Europe and one in the US (Supplementary Table 1). Leaves and ripe fruits were collected from a PPV-susceptible comparator, ‘Stanley,’ from three locations in Europe and one in the USA (Supplementary Table 1). Fruit and leaf material were shipped from Europe under Animal and Plant Health Inspection Service (APHIS) permit number P526P-11-02618 to APHIS inspection facilities before being forwarded from APHIS to the USDA, Kearneysville, West Virginia (WV) where the tissue was frozen in liquid N2, lyophilized, and stored at −20°C. Samples from field-grown trees at USDA Kearneysville were harvested and in the same manner, frozen, and stored.

All three plum cultivars named are available commercially, including ‘HoneySweet’ (https://shop.cumminsnursery.com/shop/plum-trees/honeysweet-plum). Currently, ‘HoneySweet’ has only been approved for growth and cultivation in the United States. Use in other countries will depend on the policies of that country.

### Quantification Analysis Using qPCR

Total RNA was extracted from the leaves of ‘HoneySweet’ trees (2005–2017) using Spectrum™ Plant Total RNA Kit (Sigma Aldrich, St. Louis, MO, USA) using the instructions of the manufacturer as previously described in Singh and Kundu (2017). Complementary DNA (cDNA) was synthesized using a Reverse Transcription System (Promega, Madison, WI, USA).

Quantitative PCR was performed using a real-time system LightCycler 480 (Roche, Basel, Switzerland). The titer of PPV-strain D (PPV-D) and PPV-REC was measured using specific primers for PPV-REC CP (REC-J-F–AATGATATTGATGATAGCCTTGAC, REC-J-R—AGCTGGTTGAGTTGTTGCCAC) (Jarošová et al., 2010) and PPV-D CP (PPV-FD—TCAACGACACCCGTACGGGC, PPV-RR—GGAATGTGGGTGATGATGG) (Jarošová et al., 2010) with a melting temperature at 94°C and an annealing temperature at 60°C for 35 cycles. The PCR products were visualized following electrophoresis on 1.5% agarose gel and staining by SYBR Safe (Invitrogen, Waltham, MA, USA). Standards for qPCR were prepared by cloning the target sequence into the vector pGEM®-T Easy Vector (Promega) as described earlier (Singh and Kundu, 2017).

### RNA Extraction for Sequencing

The small RNA (siRNA) from leaves and fruit (20 ng of lyophilized material) was extracted using a Norgen plant microRNA purification kit (Norgen Biotek Corp., Thorold, Canada; https://norgenbiotek.com/) following the instructions of the manufacturer. The mRNA from the fruit and leaves was extracted in the same manner.

### RNA Sequence Processing Graft-Challenged Trees

The production and sequencing of the sRNA libraries from the graft-challenged tree experiment were carried out by the Genomics Resources Core Facility (GRCF, Weill Cornell Medical College; http://corefacilities.weill.cornell.edu/). A total of 22 sRNA libraries were sequenced on an Illumina HiSeq 2500® (https://emea.illumina.com/) (Illumina, Inc., San Diego, CA, USA). The sRNA sequences were parsed from FASTAQ formatted files and adapter trimming, and cleaning of the reads was carried out using the CLC Genomic Workbench (Qiagen, Germantown, MD, USA; https://www.qiagen.com) and Cutadapt (https://cutadapt.readthedocs.io/en/stable/). Quality control of the reads was performed at each step using FASTQC implemented in the CLC Genomic Workbench. Peach genome v2.1 (chromosome 4; chr4: 25507706…258432937) was used to remove tRNA sequences from the sRNAs (The International Peach Genome Initiative, 2013). The viral genomic sequences were downloaded from NCBI (https://www.ncbi.nlm.nih.gov/). Viral siRNA (vsRNA) reads were mapped to the viral genomic sequences utilizing the CLC Genomic Workbench. Viral siRNA expression and abundance were visualized in the form of heat maps as log2-transformed reads per kilobase million (RPKM) (for sequencing) and log2-transformed quantitative data (for qPCR). The data were normalized by calculating Z-scores using the R-statistical package (Singh et al., 2014). All sRNA sequences have been deposited in GenBank, SRA, with the BioProject ID PRJNA741990.

### RNA Sequence Processing Multi-Location Trees

The mRNA and sRNA RNA-Seq libraries were constructed and sequenced by the David H. Murdock Research Institution, Kannapolis, North Carolina using an Illumina GAII sequencer and paired 75b reads. Sequences were cleaned by the trimming of adaptor sequences, Ns, and quality scores as determined by the CLC Genomic Workbench (Qiagen, USA; https://www.qiagen.com). The RNA reads were then filtered by the peach rRNA sequence, grape mitochondrial sequence, and peach chloroplast sequences with the remaining sequences mapped to the peach genome V1.0 (The International Peach Genome Initiative, 2013). The remaining sequences were then mapped to either the transgene sequences of ‘HoneySweet’ or a reconstructed PPV-D viral genome from the infected ‘Stanley’ in Spain or the reconstructed PPV-M viral genome from the infected ‘Stanley’ in Bulgaria. The reads relative to the total reads were based on the number of sequences that mapped to the peach genome after the filtering process. The mRNA sequences have been deposited in GenBank, SRA, with the BioProject ID PRJNA741796. The sRNA sequences have been deposited in GenBank, SRA, with the BioProject ID PRJNA742049.

### Mouse-Feeding Study

All procedures associated with this study were reviewed and approved by the Institutional Animal Care and Use Committee (IACUC) at Oklahoma State University (OSU). Four-week-old CD-1 female (*n* = 75) and male (*n* = 75) mice were purchased from Charles River Laboratory. Upon arrival, the mice were maintained in standard shoebox cages in the OSU environmentally controlled laboratory animal facility (12-h light/dark cycle) on a standard chow diet for 5–7 days before being randomly assigned to either the control or one of the dried plum treatment groups. The treatment groups were fed a control diet based on the American Institute of Nutrition (AIN)-93G diet (control), control-supplemented with 5 or 15% plum (w/w) from two different varieties, ‘HoneySweet’ (HS5% and HS15%) or ‘Stanley’ (ST5% and ST15%). The rationale for the doses selected is that we estimated that 5% would represent routine consumption consistent with ~25 g/day for human consumption and that the 15% would be in line with heavy consumption or ~75 g/day for humans. Dried plum is, on average, 9.5 g (https://fdc.nal.usda.gov/fdc-app.html#/food-details/168162/nutrients) so the 5% would be 2–3 dried plums and the 15% would be 7–8. The source of the ‘HoneySweet’ and Stanley plums used in the study was the Kearneysville, WV orchard. The plums were lyophilized and pooled into a batch for ‘HoneySweet’ and a batch for ‘Stanley.’ Each batch was then pulverized into a powder to have a uniform sample and then shipped to the investigators at OSU labeled as test products A and B to be incorporated into diets. The diets were formulated to have the same carbohydrate, fat, protein, fiber, calcium, and phosphorus contents, respectively (Halloran et al., 2010). Throughout the study, the mice had free access to food and reverse osmosis (RO) water. Clinical checks were performed five times per week by study personnel and included an examination of general appearance, fur, eyes, and animal behavior or activity. Food intake was documented, and body weight was recorded weekly.

At the end of the study, the mice were anesthetized with a ketamine xylazine cocktail (60 mg of ketamine and 6 mg of xylazine per kg body weight) and blood samples were collected from the carotid artery, following a 3-h fast for clinical chemistries and total white blood cell (WBC) counts before a blood smear was made for the evaluation of WBC differentials. The clinical chemistry panel and assays included the serums alanine aminotransferase (ALT), albumin (ALB), alkaline phosphatase (ALP), aspartate aminotransferase (AST), bilirubin (TBIL), blood urea nitrogen (BUN), cholesterol (CHOL), creatinine (CREAT), glucose (GLU), globulin (GLOB), total protein, (TP), triglycerides (TRIG), phosphorus (PHOS), calcium (Ca), sodium (Na), potassium (K), and chloride (Cl).

Organs and tissues were collected immediately after euthanasia and fixed in 10% neutral buffered formalin. A broad spectrum of tissues was examined, namely, the brain (representative regions: cerebrum and cerebellum), eyes, thyroid, thymus, stomach, small intestine (duodenum, jejunum, and ilium), pancreas, colon, rectum, liver, kidneys, adrenal glands, spleen, heart, aorta, trachea, lungs, gonads (testes and ovaries), uterus, vagina, female mammary glands, urinary bladder, lymph nodes, bone, and bone marrow. All tissues were processed by the OSU Pathology Laboratories in the College of Veterinary Medicine and scored based on a 0–3 scale with 0 = no apparent abnormalities, 1 = mild abnormality, 2 = moderate abnormalities, or 3 = severe abnormalities. The most common noted abnormalities were mild inflammation, which is not uncommon in mouse colonies.

The primary outcomes of this study focused on the gross and microscopic pathology of tissues, clinical chemistry, and WBC results. All statistical analyses performed on the data from the animal study utilized SAS Version 9.3 (SAS Institute, NC). For continuous variables such as body weight, blood clinical chemistries, and WBC, the effects of the treatments were compared using ANOVA and Fisher's least significant difference (LSD) *post-hoc* analyses. For categorical data such as pathology scoring and abnormal clinical chemistries, the differences in the frequency of scores were determined using the chi-square and α = 0.05 for all statistical analyses. All the investigators associated with the animal study were blinded to the treatments throughout the course of the study and data analyses. Only after a final report was generated was the code for the two different cultivars of plum revealed.

### sRNA Homologies to Mouse

The most abundant 2,834 sRNAs related to the transgenic CP sequence in ‘HoneySweet’ were used to find homologies to mouse, Mus musculus, transcripts using the Basic Local Alignment Search Tool (BLAST) and sequence database at NCBI (https://blast.ncbi.nlm.nih.gov/Blast.cgi). Matches with two or fewer mismatches and one or fewer gaps were considered significant matches.

## Results

### Efficacy of RNAi Under Intensive Long-Term Infection Pressure

#### Graft-Challenged Trees

**Figure 1:**
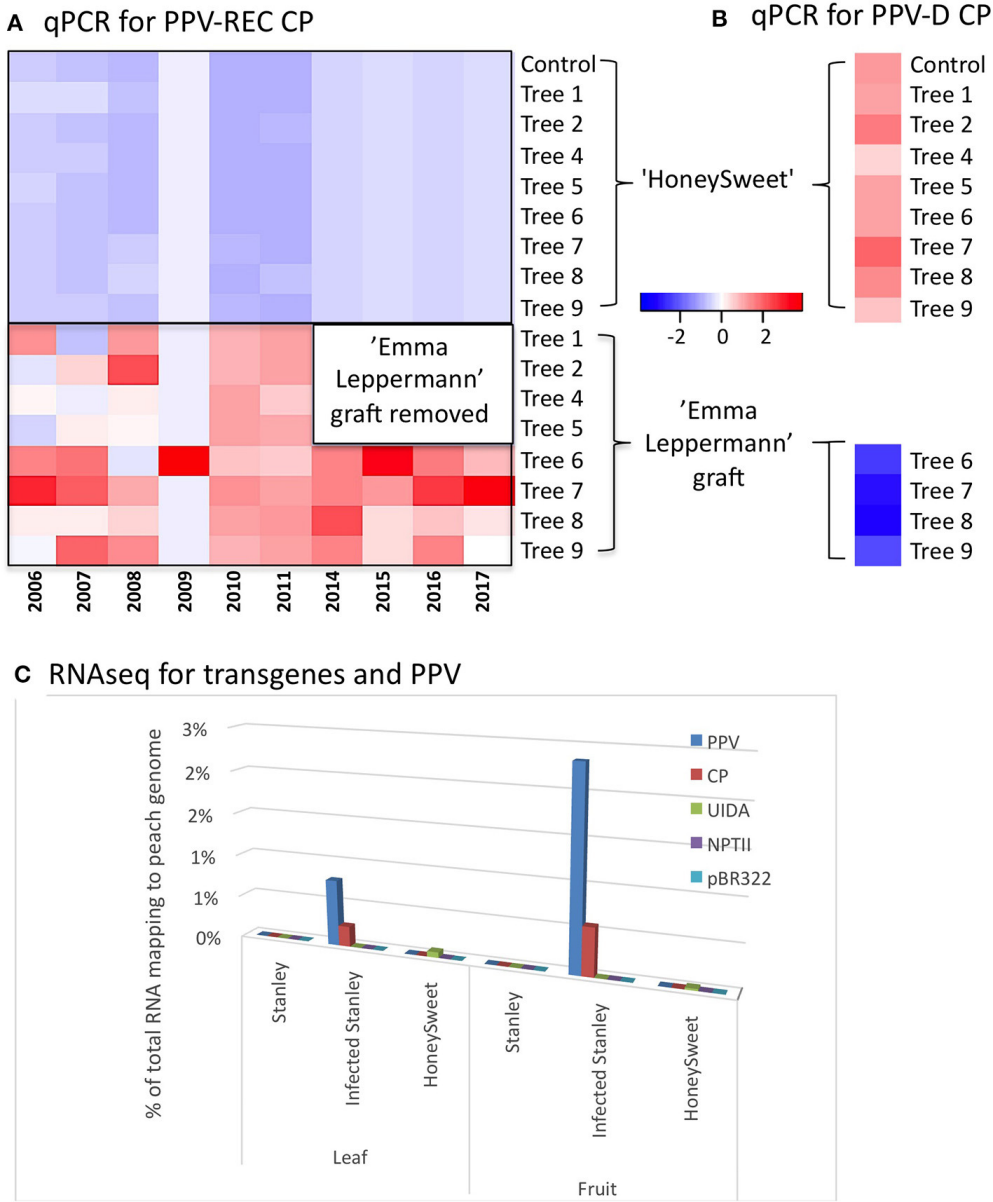
The presence or absence of PPV and expression of the transgenes in ‘HoneySweet’ and comparators, PPV-REC-infected ‘Emma Leppermann’ and ‘Stanley’ infected or not was determined by qPCR analyses for the graft-challenged material and using RNA-Seq studies for the multi-location trial. Heatmaps **(A, B)** showing the relative abundance of CP RNA in ‘HoneySweet’ trees and grafted and artificially infected ‘Emma Leppermann’ limbs. ‘HoneySweet’ contains a CP transgene derived from the PPV-D serotype and ‘Emma Lepperman’ was infected with a PPV-REC serotype (originating from Bulgarian). **(A)** Leaves from eight ‘HoneySweet’ trees and their associated grafts (infected ‘Emma Leppermann’) were sampled together with a control ‘HoneySweet’ tree with no graft, for the presence of CP RNA from PPV-REC each year using qPCR with serotype-specific primers, REC-J-F and REC-J-R. **(B)** Transgene PPV-D was quantified in the same eight ‘HoneySweet’ trees and associated grafts in four of the trees and the control ‘HoneySweet’ with no graft, for 1 year utilizing serotype-specific primers, PPV-FD and PPV-RR. Heatmaps were drawn from log-transformed (log2) using the ggplots package of the R-statistical software. Color key represents the scale distribution of the expression/abundance. The PPV-REC CP RNA was only detected in ‘Emma Leppermann’ and the PPV-D CP RNA was only detected in ‘HoneySweet’ leaves. **(C)** Amount of RNA homologous to the transgenes contained in ‘HoneySweet’ and the PPV genome, as a percentage of the total reads that map to the peach genome with those related to rRNA, mitochondrial genome, and chloroplast genome. Only the infected ‘Stanley’ tissue has a significant % reads mapping to PPV with a larger proportion found in fruit tissue.

A field trial consisting of nine ‘HoneySweet’ trees that were planted in the Czech Republic (Polák et al., 2017) was used to evaluate RNAi efficacy under long-term continual infection pressure. Eight ‘HoneySweet’ trees were inoculated with the PPV-REC *via* bud grafts from a susceptible plum variety, ‘Emma Leppermann,’ in 2002. The PPV-infected, non-transgenic grafts were allowed to grow out, producing large infected and symptomatic limbs (Supplementary Figure 2). In 2011, the infected grafts were removed from four of the trees, and all the trees were monitored for PPV infection and symptoms until 2017. The PPV-REC levels of these eight trees and a control ‘HoneySweet’ tree (not graft-challenged) were assayed by qPCR annually for 10 years in leaf tissue from ‘HoneySweet’ and the infected ‘Emma Leppermann’ graft tissue (Figure 1A). As a result, PPV-REC was detected only in the grafted non-transgenic ‘Emma Leppermann’ that had been initially infected. The PPV-D CP transgene expression was detectable in all the ‘HoneySweet’ trees and none of the ‘Emma Leppermann’ grafts (Figure 1B). The results show that, even under intense infection pressure from grafted infected limbs, the ‘HoneySweet’ trees remained virus-free after over a decade of continuous virus exposure.

#### Naturally Infected Tree Multilocation

‘HoneySweet’ leaf and ripe fruit tissue samples were collected from mature trees in field test plots located in Spain, the Czech Republic, and from the United States where there was no possibility of PPV exposure. Samples of ‘Stanley’ were collected at the same or similar sites as a comparator. ‘Stanley’ is highly susceptible to PPV but is grown commercially in Europe as it displays few symptoms in infected fruit. Among the 10 ‘Stanley’ trees sampled, 2 ‘Stanley’ trees from Spain were found to be infected with the PPV-D strain while 2 ‘Stanley’ trees from Bulgaria had been infected with the PPV-M strain based on positive ELISA results (data not shown). The reads quantified from RNA-Seq and sRNA-Seq were also found to be matching their respective PPV strain genomes (Figure 1C; Supplementary Table 2). After over 8 years of exposure to endemic PPV in Spain and the Czech Republic, none of the ‘HoneySweet’ trees had detectable PPV. The levels of virus and transgene RNA relative to total mRNAs are shown in Figure 1C and Supplementary Table 2. The amount of transgene CP mRNA was less than 1/100 of the amount of natural virus CP RNA in the infected ‘Stanley’ trees. Interestingly, the amount of viral RNA was ~two-fold higher in fruit than in the leaves of the naturally infected ‘Stanley’ trees even though leaves expressed symptoms of PPV infection while fruits generally did not. For ‘HoneySweet,’ there were approximately equal amounts of CP transgene RNA in leaves and fruit relative to total mRNA. The levels of sRNAs related to PPV and transgenes reflected the same profile trends as the mRNAs (Supplementary Table 2).

In summary, we found that the transmission of PPV to ‘HoneySweet’ was not detected after more than 8 years in the field, and the relative levels of transgene RNA were considerably less than that of viral CP RNA found in the infected, susceptible ‘Stanley’ trees.

### sRNA Profiling of Transgene

#### Size Distribution of sRNA

Total sRNAs were sequenced for the graft-challenged ‘HoneySweet,’ the infected graft material ‘Emma Leppermann,’ and the multiple locations ‘HoneySweet’ and ‘Stanley’ samples. The RNAs that had homology to the CP transgene and the whole PPV sequence were sorted by size. The most striking differences were in the higher amount of 22 nt RNAs in infected ‘Stanley’ and the higher amount of 24 nt RNAs in ‘HoneySweet.’ All the ‘HoneySweet’ samples had similar proportions of sRNA by size, namely, 21 nt sRNAs (50–70% total), >22 nt sRNAs (20–30% total), >24 nt sRNAs (5–15% total), and the negligible detections of other sizes (Supplementary Figure 3). There were no differences between trees exposed and unexposed to PPV (Supplementary Figure 3). There was a small difference in the distribution between ‘HoneySweet’ leaves and fruits, in that fruit had an average of 23% of sRNAs in the 24-nt class while leaf had ~9%. No significant differences were observed between the fruits and leaves in infected ‘Stanley’ trees. Likewise, the infected graft material, ‘Emma Leppermann,’ had similar amounts of 21 nt sRNAs, somewhat higher levels of 22 ng sRNAs, and negligible amounts of the other species including 24 nt sRNAs (Supplementary Figure 3).

#### Abundance of the Transgene- and Viral-Related sRNAs

**Figure 2:**
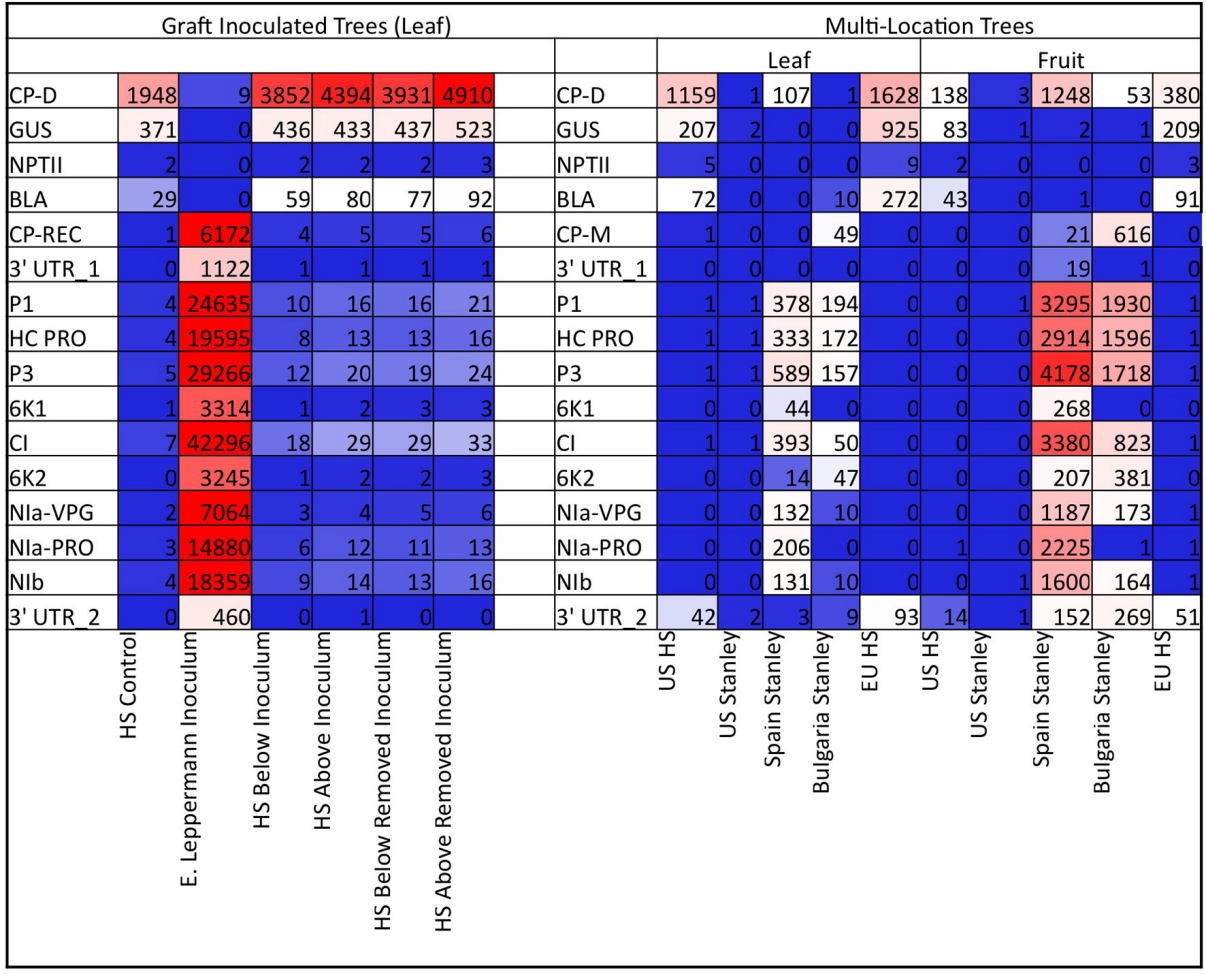
Heatmap of the abundance of sRNAs with homologies to transgenes and PPV viral genes. The number of sRNA reads that matched transgenes (CP-D, GUS, NPTII, BLA) or PPV genes (CP-REC or CP-M, 3′UTR_1, P1, HC-PRO, P3, 6K1, CI, 6K2, Nla-VPG, Nla-PRO, Nib, 3′UTR_2) were tabulated and standardized by amounts per 1 million total sRNA reads. The higher the rate of reads, the more red, and fewer, including no reads, the more blue. The actual number of reads per million is included in each box. The values are an average of the trees under each category. The first set represents the graft-introduced PPV-REC. The six categories are HS control (uninfected ‘HoneySweet,’ one tree), E. Leppermann (‘Emma Leppermann’ infected grafts, four trees), HS below inoculum (‘HoneySweet,’ four trees where leaves were collected below the infected graft), HS above inoculum (‘HoneySweet,’ four trees where leaves were collected above the infected graft), HS below removed inoculum (‘HoneySweet,’ four trees where leaves were collected below where the infected graft had been), and HS above removed inoculum (‘HoneySweet,’ four trees where leaves were collected above where the infected graft had been). The second group is the multi-location ‘HoneySweet’ and ‘Stanley’ (infected and not). The 10 categories are US HS (‘HoneySweet,’ three trees with no PPV exposure), US Stanley (‘Stanley,’ 3 trees with no PPV exposure), Spain Stanley (‘Stanley,’ two trees, positive for PPV-D infection), Bulgaria Stanley (‘Stanley,’ two trees positive for PPV-M infection), and EU HS (‘HoneySweet’ from Spain and the Czech Republic, five trees). Both leaf material and fruit material were analyzed for the related species of sRNA. The map points out that in the graft-challenged experiment, basically only the initially infected ‘Emma Leppermann’ grafts contained sRNAs related to the virus and in the multi-location experiment, the infected ‘Stanley,’ both leaf and fruit were the only ones that had sRNAs related to the virus. In both experiments, ‘HoneySweet’ had sRNAs related to the transgenes, primarily CP and GUS.

To assess the potential risks posed by sRNAs, specific species of sRNAs that matched transgene sequences and viral sequences were quantified. In the graft-challenged ‘HoneySweet,’ the sRNAs in both the ‘HoneySweet’ and PPV-infected ‘Emma Leppermann’ were tabulated including the sRNA related to each transgene (*CP, UIDA, NPT*, and *BLA*) and each gene of PPV-REC (Figure 2). The levels of sRNA derived from the CP transgene in ‘HoneySweet’ were generally lower than those homologous to the CP from the PPV-REC in infected ‘Emma Leppermann’ leaves based on total read counts (Figure 2; Supplementary Data Sheet 1: Graft Challenged). In the case of the ‘HoneySweet’ and ‘Stanley’ from different locations, a similar pattern was observed in that viral RNAs were found in the infected ‘Stanley’ leaves and, to a greater extent, in fruits. Also, similar to the graft-challenged experiment, there were more CP-related sRNAs in trees that were exposed to PPV yet had no detectable virus RNA (Figure 2; Supplementary Data Sheet 2: Multi-location).

#### Consistency of sRNA Profiles

The eight individual trees that were graft-challenged in the Czech Republic along with the multiple-environment tree planting allowed us to look at the variation of CP sRNA in ‘HoneySweet’ trees under different cultivation practices, soils, and climates (Supplementary Data Sheet 3, ANOVA analyses). While there was variation among trees at the same location, there was only a statistically significant variation in the CP sRNA between fruit and leaf tissues and between ‘HoneySweet’ fruit grown in the US over different years and fruit grown at two locations in Europe for 1 year.

#### Relative Abundance of Transgene sRNAs

We noted that the relative abundance of individual sRNA species derived from the CP transgene in ‘HoneySweet’ was significantly lower than many of the endogenous sRNAs. When ranked by expression level, the most abundant CP-specific sRNA species ranked 11,007 out of all identified sRNAs with chloroplast-, ribosomal-, and miRNA-related species from 10 to 100-fold higher levels of expression (data not shown). This suggests that the 35S hairpin cassette present in the ‘HoneySweet’ genome does not produce high levels of sRNA in leaves or fruits relative to native sRNA species.

#### Movement of Transgene and Viral sRNAs Between ‘HoneySweet’ and Grafted PPV-Infected ‘Emma Leppermann’

We were able to assess if the virus and/or virus sRNAs moved from the infected graft into ‘HoneySweet’ or if transgene-derived sRNAs moved into the infected graft because of the sequence differences between the transgenes and PPV-REC (Supplementary Data Sheet 1). Leaf samples were collected from ‘HoneySweet’ both close to the graft site (lower) and further from the graft site in the upper portion of the tree to consider potential limited distance mobility (Supplementary Figure 2). Samples were also collected from the grafted ‘Emma Leppermann.’ No statistically significant levels of transgene-specific sRNA were found in ‘Emma Leppermann’ and little to no viral sRNAs were found in ‘HoneySweet.’ Furthermore, no significant differences were detected between the samples taken below or above the graft union (Figure 2; Supplementary Data Sheet 1). Thus, no evidence of sRNA translocation from ‘HoneySweet’ into the infected ‘Emma Leppermann’ graft was found. The presence of low levels of viral sRNA in ‘HoneySweet’ could represent either translocated sRNAs or a consequence of the action of RNAi machinery on the infecting virus.

### Lack of Transgene-Induced Selection Pressure on Virus Populations

**Figure 3:**
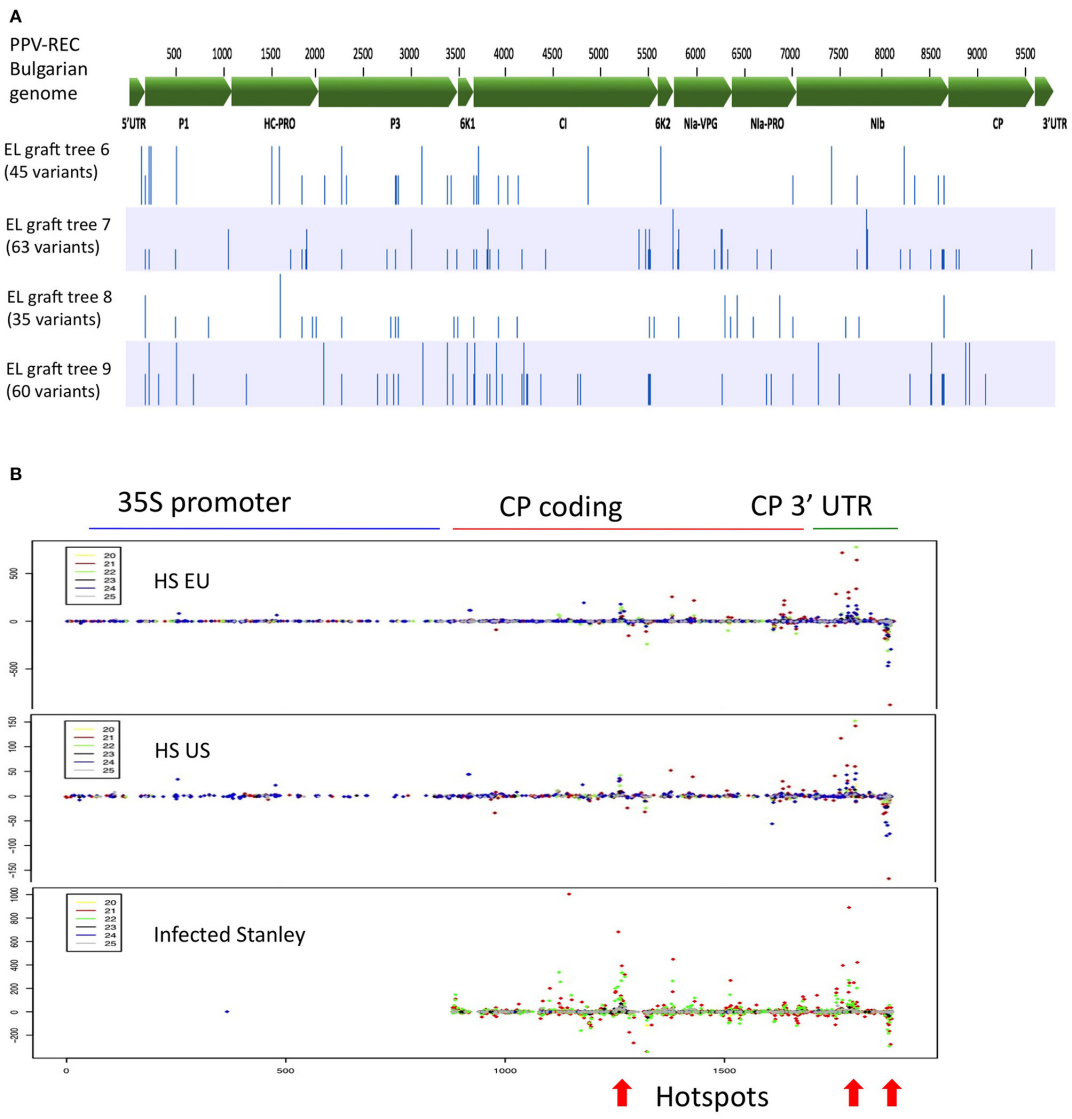
Distribution of mutations in the PPV-REC genome after 10 years of exposure and the distribution of sRNAs related to the CP gene after 9 years of growth. **(A)** Sequence variation of the viral PPV-REC genome found in four different grafts of ‘Emma Lepperman’ on ‘HoneySweet’ for over 10 years. Variant analysis was performed by aligning viral sRNA reads (vsRNA) to the whole genome of PPV-REC strain of PPV including 5′ and 3′ UTRs. Genes and their positions on the genome are marked on the top with a green bar while variants are marked with blue bars for the individual tree. **(B)** Fruit sRNAs were mapped to the PPV-CP and promoter sequence of the transgene in ‘HoneySweet’ or the PPV CP in ‘Stanley.’ Each block represents the sRNAs from European-grown ‘HoneySweet’ (HS EU), United States-grown ‘HoneySweet’ (HS US), and ‘Stanley’ naturally infected by PPV. Each color represents a different length of sRNA (yellow-20 nt, red-21 nt, green-22 nt, black-23 nt, blue-24 nt, gray-25 nt). Dots above the bar represent the number of positive-strand sRNAs and those below represent the number of negative strand sRNAs. There are several regions of sequence that have larger numbers of related sRNAs, so-called hotspots. These can be seen in all three conditions, including the naturally infected ‘Stanley,’ and are indicated by an arrow at the bottom of the figure.

We assessed whether there was any evidence of evolutionary pressure or adaptive evolution imposed by the ‘HoneySweet’ RNAi mechanism on PPV-REC present in the grafted ‘Emma Leppermann’ tissues. We reconstructed the PPV-REC genomic sequences from each infected tree by assembling the viral sRNA reads against the PPV-REC reference genome and identified the nucleotide variants present in each sample. The full list of synonymous and non-synonymous nucleotide variants is given in Supplementary Data Sheet 4. We observed a total of 131 unique single nucleotide polymorphisms (SNPs) in all samples (30–50 per sample) that were distributed throughout the PPV-REC genome (Figure 3A). The SNPs at 21 positions of the viral genome were present in more than one sample and, in all but two cases, the substitutions were the same. The SNPs showed a non-even distribution across the genome but relatively very few within the CP open reading frame (ORF), which is targeted by the ‘HoneySweet’ transgene.

### Non-uniform Distribution of sRNA Produced From the Transgene

We examined the production of sRNA along with the CP in both ‘HoneySweet’ and virus-infected ‘Stanley’ and also found it to be non-uniform, consisting of “hotspots” where most of the identified sRNA species were derived. This was the case for US-grown ‘HoneySweet,’ European-grown ‘HoneySweet,’ and infected ‘Stanley’ (Figure 3B). There appeared to be islands of sequences that have different sized sRNAs overlapping in sequence. Interestingly, the sRNA species within the hotspots were highly variable among different samples, tissues, and virus strains, suggesting that they were not due to a sequence-specific sRNA ligation bias during the library preparation, but instead represent regions producing higher sRNA levels (Figure 3B, Supplementary Table 3).

### Dose-Dependent Effects of ‘HoneySweet’ Plum Consumption and General Clinical Signs in Mice

#### Weight Gain in Response to Diets Containing Plum

**Figure 4:**
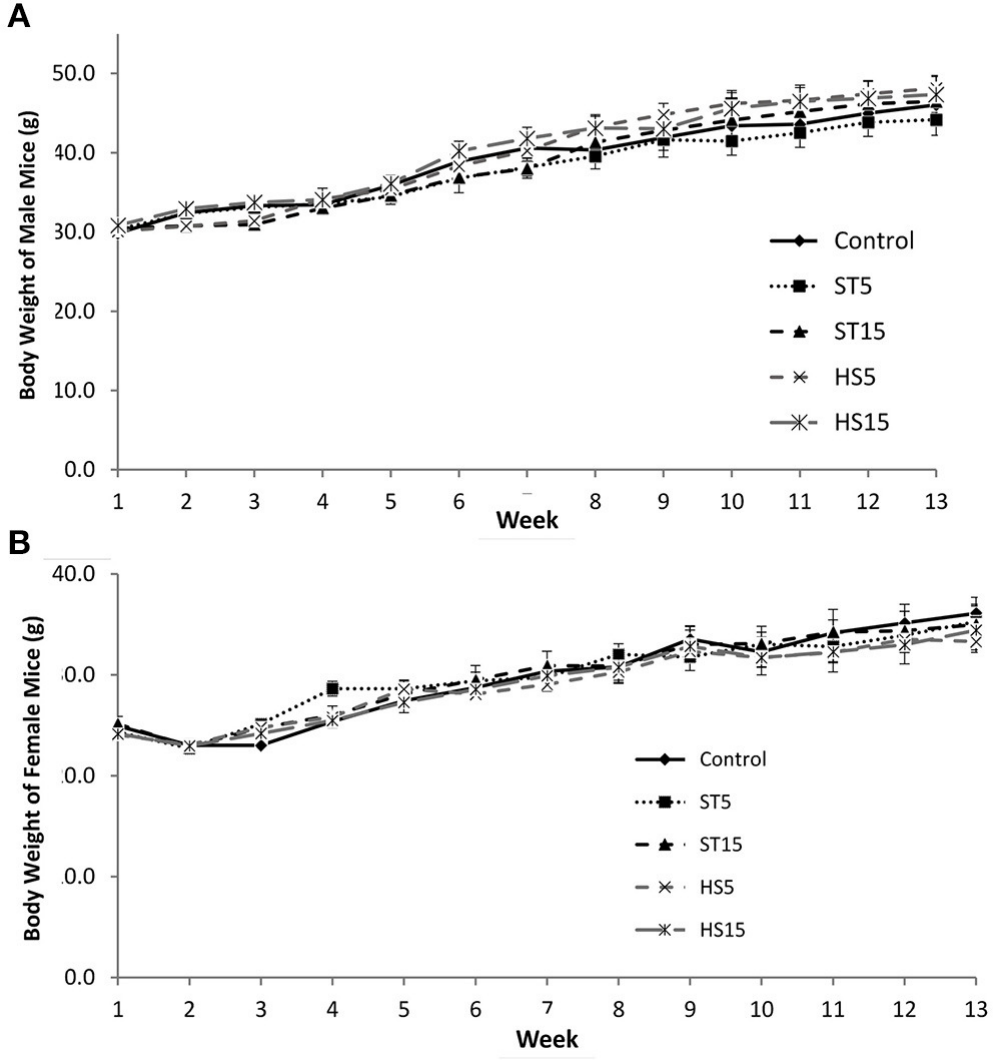
Comparison of weight gain of mice fed either transgenic ‘HoneySweet’ dried fruit, ‘Stanley’ dried fruit or a control diet. **(A)** Body weight of male (*n* = 75), CD-1 mice. **(B)** Body weight of female (*n* = 75), CD-1 mice. No significant differences were observed in body weight across the 90-day study duration. Error bars reflect SE. Control = standard mouse diet; ST5, standard mouse diet with 5% by weight ‘Stanley’ dried plum included; ST15, standard mouse diet with 15% by weight ‘Stanley’ dried plum included; HS5, standard mouse diet with 5% by weight ‘HoneySweet’ dried plum included, and ST15, standard mouse diet with 15% by weight ‘HoneySweet’ dried plum included.

To assess the potential health impacts of ‘HoneySweet’ sRNA consumption, we performed a comprehensive 90-day feeding study in outbred CD-1 mice. The experimental design included both male and female mice, two doses of ‘HoneySweet’ plum (HS5% or HS15% of diet) along with two doses of non-transgenic uninfected ‘Stanley’ fruit (ST5% or ST15%), and an AIN-93 control diet (CD) lacking plum. The body weights of all mice were recorded on a weekly basis, and no effects of either ‘Stanley’ or ‘HoneySweet’ plum consumption at either dose were observed in either the male (Figure 4A) or female (Figure 4B) mice at any time point throughout the 90-day study period. Likewise, neither change in body weight nor percent change in body weight were affected by the sample type or the dose over the study period (data not shown). Food intake was recorded throughout the study, and no differences in food intake were documented between treatment groups in either gender nor were any noticeable clinical alterations in animal behavior observed in terms of grooming and physical activity (data not shown).

#### Effects of Consumption of Plum on White Blood Cells, Clinical Chemistries, and Pathology

To assess the general physiological functions in the mice, blood chemistries were performed on sera collected at the end of the study. Of the analytes assessed, only the triglycerides, total protein, and BUN revealed significant differences between treatment groups, but only in the male cohort (Table 1). The ‘Stanley’ plum 15% dose (ST15%) exhibited the greatest increase in serum triglycerides compared to the CD and ST5% groups. Triglycerides were also elevated in the HS15% and HS5% groups, but no dose-dependent effects were observed. Serum total protein was reduced in the HS5% group compared with the controls, but all values remained within the normal range. Likewise, differences existed between groups in terms of BUN, with the groups consuming ST5%, HS5%, and HS15% exhibiting a lower serum BUN compared with the CD group. However, all values were within the normal range. Within the female cohort of mice, no alterations in any blood chemistries were observed in response to the ‘HoneySweet’ plum or ‘Stanley’ plum and all values were within the normal limits (Supplementary Table 4).

**Table 1:** ‘HoneySweet’ study blood chemistry for males.

**Parameter**	**Control**	**ST 5%^1^**	**ST 15%^2^**	**HS 5%^3^**	**HS 15%^4^**	***P*** **value**	**Normal range^5^**
Chol^6^ (mg/dL)	142.64 ± 9.53	147.09 ± 4.62	162.50 ± 7.34	149.83 ± 9.90	146.00 ± 8.12	0.4778	–
TRIG (mg/dL)	113.82 ± 9.24^cd^	127.18 ± 10.42^bd^	167.08 ± 14.13^a^	154.83 ± 14.85^ab^	150.25 ± 13.62^ac^	**0.0351**	–
ALT (U/L)	27.42 ± 5.30	45.82 ± 13.46	74.67 ± 51.52	40.92 ± 6.13	38.50 ± 6.74	0.7177	17–77
AST (U/L)	86.75 ± 18.31	104.64 ± 21.63	103.08 ± 33.78	114.67 ± 23.15	86.75 ± 8.66	0.8813	54–298
GLU (mg/dL)	276.45 ± 24.24	228.45 ± 25.42	256.83 ± 30.71	194.33 ± 16.50	220.67 ± 30.93	0.2193	62–300
PHOS (mg/dL)	9.36 ± 0.39	9.94 ± 0.6	10.56 ± 1.02	10.82 ± 1.52	8.12 ± 0.25	0.2427	5.7–9.2
Ca (mg/dL)	9.08 ± 0.11	9.06 ± 0.12	9.31 ± 0.05	8.99 ± 0.11	8.97 ± 0.06	0.0893	7.1–10.1
TBIL (mg/dL)	0.24 ± 0.03	0.20 ± 0.01	0.20 ± 0.01	0.18 ± 0.01	0.20 ± 0.02	0.2464	0–0.9
TP (g/dL)	4.60 ± 0.06^abc^	4.58 ± 0.08^bcd^	4.64 ± 0.03^ac^	4.41 ± 0.05^d^	4.43 ± 0.10^de^	**0.0470**	3.5–7.2
ALB (g/dL)	2.57 ± 0.10	2.62 ± 0.07	2.69 ± 0.08	2.39 ± 0.08	2.48 ± 0.08	0.1080	2.5–3.0
GLOB (g/dL)	1.94 ± 0.05	1.97 ± 0.03	1.89 ± 0.03	1.90 ± 0.04	1.86 ± 0.05	0.3713	1.0–4.2
AG ratio	1.39 ± 0.04	1.37 ± 0.03	1.46 ± 0.04	1.33 ± 0.04	1.36 ± 0.04	0.2695	–
BUN (ng/dL)	26.64 ± 1.71^a^	19.91 ± 1.01^bc^	25.08 ± 0.65^a^	17.25 ± 0.46^c^	21.00 ± 1.40^b^	**0.0001**	8–33
CREAT (mg/dL)	0.22 ± 0.01	0.21 ± 0.01	0.20 ± 0.00	0.20 ± 0.00	0.20 ± 0.00	0.2833	0.2–0.9
Na (mEq/L)	144.82 ± 0.48	145.90 ± 0.84	145.18 ± 1.08	144.10 ± 0.62	145.82 ± 0.92	0.5359	140–160
K (mEq/L)	5.22 ± 0.21	5.06 ± 0.12	5.05 ± 0.12	5.98 ± 1.04	4.67 ± 0.17	0.3836	5–7.5
CL (mEq/L)	110.27 ± 0.56	110.70 ± 0.94	110.09 ± 1.14	109.40 ± 1.18	110.91 ± 0.55	0.7986	88–110
Na/K	28.21 ± 1.13	28.00 ± 0.72	28.90 ± 0.78	27.47 ± 2.28	31.65 ± 1.27	0.2381	–

*Treatments were compared using an ANOVA and Fisher's least significant difference (LSD)*.

*Only three measurements had a p-value of 0.05 or lower and these are highlighted in bold*.

*Those values are then grouped as statistically different classes by the small superscript letters, abcde. Where classes overlap, they have two or more letters*.

1 *‘Stanley’ was added at 5% of the total weight of the diet*.

2 *‘HoneySweet’ was added at 5% of the total weight of the diet*.

3 *‘Stanley’ was added at 15% of the total weight of the diet*.

4 *‘HoneySweet’ was added at 5% of the total weight of the diet*.

5 *Where known, the normal range of components found in the blood. Where blank, there is no consensus level*.

6 *ALT, alanine aminotransferase; ALB, albumin; ALP, alkaline phosphatase; AST, aspartate aminotransferase; TBIL, bilirubin; BUN, blood urea nitrogen; CHOL, cholesterol; CREAT, creatinine; GLU, glucose; GLOB, globulin; TP, total protein; TRIG, triglycerides; PHOS, phosphorus; Ca, calcium; Na, sodium; K, potassium; Cl, chloride*.

Total WBC counts and differentials were assessed as general indicators of the immune response to plum. In the male animals, there were no differences in total WBC, mean percent or absolute neutrophils, monocytes, eosinophils, and basophils counts (Supplementary Table 5). Statistically significant differences were detected in the percent lymphocytes between the two cultivars of plum at the 5% dose (i.e., ST5% and HS5%) and the percent of lymphocytes in the HS5% was significantly higher than the CD group, but they were still within the normal range of values. Among the female cohort, the percent neutrophils were lower in the ST5% and ST15% treatment groups compared with the HS15% group, but again, total WBC and the percent and absolute differential counts were within the normal range (Supplementary Table 6). These findings indicate that no negative effects of plum on circulating leukocyte populations were detected.

An evaluation of the 38 different organs and tissues commonly evaluated in toxicology studies was performed representing all major physiological systems. The team of pathologists performing the evaluations were blinded to treatments throughout the course of reading and scoring the slides. In the male cohort, there were no differences between the groups in terms of the pathology scores for any of the organs and tissues assessed, indicating no negative effects of the ‘HoneySweet’ plum at either dose (Table 2). Mild salivary gland sialadenitis and the peripelvic nephritis of the kidney were noted in all groups including the CD mice, but there were no specific effects associated with ‘HoneySweet’ plum consumption. For the female cohort, similar observations were made with no apparent differences in pathology scores among any of the groups, including the HS5% and HS15% groups (Supplementary Table 7). The female animals exhibited a similar mild inflammation within the salivary glands and kidneys, but there was also a mild hepatic inflammation and vacuolation within the adrenal glands that was present to some degree in all groups. In summary, these data show that no detrimental effects of ‘HoneySweet’ plum consumption were detected.

**Table 2:** The pathology report for male mice fed normal mouse chow or chow supplemented with either ‘Stanley’ plum at 5 and 15% or ‘HoneySweet’ plum at 5 and 15%.

**Tissue**	**Control**	**ST^a^5%**	**ST15%**	**HS^b^5%**	**HS15%**	**Chi Sq (***P*** value)**
Heart	0/12^c^(0%)	0/12 (0%)	1/12 (8.3%)	0/12 (0%)	0/12 (0%)	0.397
Aorta	0/12 (0%)	0/11 (0/5)	1/12 (8.3%)	0/11 (0/5)	0/11 (0/5)	0.431
Lung	1/12 (8.3%)	0/12 (0%)	0/12 (0%)	0/12 (0%)	0/12 (0%)	0.397
Trachea	0/12 (0%)	0/12 (0%)	0/12 (0%)	0/8 (0%)	0/12 (0%)	–
Thyroid	0/7 (0%)	2/10 (20%)	0/10 (0%)	0/5 (0%)	0/8 (0%)	0.177
Thymus	0/11 (0/5)	0/12 (0%)	0/12 (0%)	0/11 (0/5)	0/10 (0%)	–
Lymph node	0/10 (0%)	0/7 (0%)	0/10 (0%)	0/9 (0%)	0/11 (0/5)	–
Bone	0/12 (0%)	1/12 (8.3%)	0/12 (0%)	0/12 (0%)	0/12 (0%)	0.397
Bone marrow	0/12 (0%)	0/12 (0%)	0/12 (0%)	0/12 (0%)	0/12 (0%)	–
Skeletal muscle	0/12 (0%)	0/12 (0%)	0/12 (0%)	0/12 (0%)	0/12 (0%)	–
Cerebrum-Gray	0/12 (0%)	0/12 (0%)	0/12 (0%)	0/12 (0%)	0/12 (0%)	–
Cerebrum-White	0/12 (0%)	0/12 (0%)	0/12 (0%)	0/12 (0%)	0/12 (0%)	–
Cerebellum	0/12 (0%)	0/12 (0%)	0/12 (0%)	0/12 (0%)	0/12 (0%)	–
Meninges 1 and 2	0/12 (0%)	0/12 (0%)	0/12 (0%)	0/12 (0%)	0/12 (0%)	–
spinal Cord	0/12 (0%)	0/12 (0%)	0/12 (0%)	0/12 (0%)	0/12 (0%)	–
Peripheral Nerve	0/12 (0%)	0/12 (0%)	0/12 (0%)	0/12 (0%)	0/12 (0%)	–
Eye-LENS	0/12 (0%)	0/12 (0%)	0/12 (0%)	0/12 (0%)	0/11 (0/5)	–
Eye-UVEA	0/12 (0%)	0/12 (0%)	0/11 (0/5)	0/12 (0%)	0/12 (0%)	–
Eye-Retina	0/12 (0%)	0/12 (0%)	0/10 (0%)	0/11 (0/5)	0/12 (0%)	–
Eye-Cornea	0/12 (0%)	0/12 (0%)	0/12 (0%)	0/12 (0%)	0/12 (0%)	–
Testis	0/12 (0%)	0/12 (0%)	0/12 (0%)	0/12 (0%)	0/12 (0%)	–
Acc. Sex Gland	0/12 (0%)	0/12 (0%)	0/12 (0%)	0/12 (0%)	0/12 (0%)	–
Skin	2/12 (16.7%)	0/12 (0%)	0/12 (0%)	0/12 (0%)	0/12 (0%)	0.082
Liver	0/12 (0%)	0/12 (0%)	0/12 (0%)	0/12 (0%)	0/12 (0%)	–
Kidney^d^	3/12 (25%)	1/12 (8.3%)	2/12 (16.7%)	3/11 (27.3%)	3/12 (25%)	0.77
Urinary bladder	0/12 (0%)	0/12 (0%)	0/12 (0%)	0/12 (0%)	0/12 (0%)	–
Spleen	0/12 (0%)	0/12 (0%)	0/12 (0%)	0/12 (0%)	0/12 (0%)	–
Salivary glands^e^	3/12 (25%)	1/12 (8.3%)	3/12 (25%)	1/12 (8.3%)	2/12 (16.7%)	0.581
Esophagus	0/12 (0%)	0/12 (0%)	0/12 (0%)	0/12 (0%)	0/12 (0%)	–
Stomach	1/12 (8.3%)	1/12 (8.3%)	2/12 (16.7%)	1/12 (8.3%)	2/12 (16.7%)	0.914
Pancreas	0/12 (0%)	0/12 (0%)	0/12 (0%)	1/12 (8.3%)	1/12 (8.3%)	0.541
Duodenum	0/12 (0%)	0/12 (0%)	0/12 (0%)	0/12 (0%)	0/12 (0%)	–
Jejunum	0/12 (0%)	0/12 (0%)	0/12 (0%)	0/12 (0%)	0/12 (0%)	–
Ileum	0/12 (0%)	1/12 (8.3%)	1/12 (8.3%)	0/12 (0%)	0/12 (0%)	0.541
Cecum	0/12 (0%)	0/12 (0%)	0/12 (0%)	0/12 (0%)	0/12 (0%)	–
Colon	0/12 (0%)	0/12 (0%)	0/12 (0%)	0/12 (0%)	0/12 (0%)	–
Rectum	0/12 (0%)	0/12 (0%)	0/12 (0%)	0/12 (0%)	0/12 (0%)	–
Adrenal glands	0/11 (0/5)	0/9 (0%)	0/12 (0%)	0/11 (0/5)	0/10 (0%)	–

*There were no negative effects seen that could be attributed to the presence of the ‘HoneySweet’ plum*.

a *ST, ‘Stanley*.’

b *HS, ‘HoneySweet*.’

c *Data presented as the number of animals with any abnormal pathology per tissue read*.

d *Salivary glands have mild sialadenitis*.

e *Kidneys have mild peripelvic nephritis*.

### Potential for Animal Off-Targeting by ‘HoneySweet’ sRNA Species

To predict the potential off-target effects of the most abundant sRNA species, we BLAST-searched individual sRNA sequences (most abundant 240 sRNAs) related to the transgenes in ‘HoneySweet’ against the mouse genome (Table 3; Supplementary Data Sheet 5). Among the mouse genes identified as potential matches were myoferlin (*Myof*), 5'nucleotidase ecto (*Nt5e*), and low-density lipoprotein receptor-related protein 6 (*Lrp6*). In particular, *Myof* encodes for a member of the ferlin family of proteins, which functions in muscle tubules and has been implicated in myoblast fusions within muscle tissue (Davis et al., 2002). Mice were deficient in this protein exhibit defects in muscle regeneration and angiogenesis. The *Nt5e* gene, also known as *CD73*, encodes for the membrane-bound nucleotidase, which hydrolyzes extracellular nucleoside monophosphates. Mice that lack this protein exhibit the calcification of joints and arteries, and alterations in the dendritic cell infiltration in tissues (Rashdan et al., 2016). Furthermore, *Lrp6* encoded protein functions as the receptor or in conjunction with *frizzled* as a co-receptor for *Wnt*, thereby activating the canonical *Wnt*/beta-catenin signaling pathway (He et al., 2004). Through the *Wnt*/beta-catenin signaling cascade, this gene is involved in the regulation of cell differentiation, proliferation, and migration. Mutations in *Lrp6* have been associated with diseases such as Alzheimer's, cancer, and osteoporosis (van Meurs et al., 2008; Liu et al., 2010, 2014). Potential pathologies associated with the altered expression of the off-target genes *MYOF, NT5E*, or *LRP6* were also assessed. No evidence of muscle dysfunction or histological anomalies was reported in the skeletal muscles of any of the animals in the study. Likewise, there were no alterations in lesions consistent with Alzheimer's in brain tissue sections, and no increased incidence of neoplasm or abnormal bone phenotypes were noted in any of the pathological reports.

**Table 3:** PPV-CP sRNAs best match *Mus musculus*.

	**Raw**		**Normalized TPM**				**Match**		**Bases**				
	**Rank^a^**	**Reads^b^**	**HSF^c^**	**STF^d^**	**HSL^e^**	**STL^f^**	**Length**	**Accession#**	**Matched**	**Gap**	**Start**	**Stop**	**Identity**
AGAGGACACAGAGAGACACACC	17	1219	22.43	9.45	26.81	–	22	gi|315075272|	20	1	1	20	Low density lipoprotein receptor-related protein 6 Lrp6
								ref|NM_008514.4|					
AGAGGACACAGAGAGACACAC	27	799	17.15	–	18.47	–	21	gi|315075272	20	1	1	20	Low density lipoprotein receptor-related protein 6 Lrp6
								|ref|NM_008514.4|					
AGGGGAGTGTAGTGGTCTCGG	89	293	3.06	0.89	8.77	–	21	gi|153791795|	19	1	3	21	Myoferlin Myof
								ref|NM_001099634.1|					
GGGGAGTGTAGTGGTCTCGGT	149	170	6.65	1.52	1.05	–	21	gi|153791795	20	1	2	21	Myoferlin Myof
								|ref|NM_001099634.1|					
AGGGGAGTGTAGTGGTCTCGGT	150	169	1.37	–	5.57	–	22	gi|153791795|	20	1	3	22	Myoferlin Myof
								ref|NM_001099634.1|					
GGTGTGTCTCTCTGTGTCCT	211	103	4.27	1.07	0.39	–	20	gi|284004882|	18	0	1	18	Tripartite motif-containing 66 Trim66
								ref|NM_001170913.1|					
GGTGTGTCTCTCTGTGTCCT	211	103	4.27	1.07	0.39	–	20	gi|284004880	18	0	1	18	Tripartite motif-containing 66 Trim66 variant 1
								|ref|NM_001170912.1|					
GGTGTGTCTCTCTGTGTCCT	211	103	4.27	1.07	0.39	–	20	gi|284004878|	18	0	1	18	Tripartite motif-containing 66 Trim66 variant 2
								ref|NM_181853.4|					
TAAAAATCAAAGGCATATCTG	139	187	0.63	1.96	5.96	–	21	gi|118130656|ref|NM_029775.2|	19	0	3	21	DCN1 defective in cullin neddylation 1 domain containing 5 Dcun1d5
AGAGCTCCGCAGTCTTGTTT	184	129	–	5.08	2.81	–	20	gi|158341626|	18	1	1	18	Phospholipase B domain containing 2 (Plbd2)
								ref|NM_023625.4|					
AAATGACTTCAACGACACCCG	193	119	0.90	0.98	3.55	–	21	gi|22208851|	19	1	1	19	Olfactory receptor142 OOlfr142
								ref|NM_146984.1|					
GCAACCTGACAGACTACAGCC	223	93	0.63	2.05	2.26	–	21	gi|118150646|	19	1	1	19	RNAexonuclease 1 homolog
								ref|NM_025852.3|					
GTTCCATTCTCTATGCACCAA	114	227	0.42	–	8.53	–	21	gi|142349713|	19	1	2	20	Member RAS oncogene RAB9B
								ref|NM_176971.2|					
CTTTTAGACAAATTATGGCAC	155	161	–	2.85	5.03	–	21	gi|256985127|	19	2	2	20	CCR-4-NOT transcription complex, subunit4 (Cnot4) variant 3
								ref|NM_001164411.1|					
CTTTTAGACAAATTATGGCAC	155	161	–	2.85	5.03	–	21	gi|256985125|	19	2	2	20	CCR-4-NOT transcription complex, subunit4 (Cnot4) variant 2
								ref|NM_016877.4|					
CATTTCTCAATGCTGCTGCCT	225	92	–	–	3.58	–	21	gi|291045287|	19	1	1	19	5′ nucleotidase ecto (Nt5e)
								ref|NM_011851.4|					
CATTTCTCAATGCTGCTGCCT	225	92	–	–	3.58	–	21	gi|141802310|	19	1	1	19	Coiled-coil domain containing 68 Ccdc68
								ref|NM_201362.2|					

*The most abundant CP-specific sRNAs were matched to all cDNA sequences from the mice to determine if there were significant homologies*.

a *Rank ordered by most abundant sRNA related to CP*.

b * Total reads for 16 ‘HoneySweet’ libraries*.

c *‘HoneySweet’ fruit*.

d *‘Stanley’ fruit*.

e *‘HoneySweet’ leaf*

f *‘Stanley’ leaf*.

## Discussion

The recent decision by the EU to regulate gene-edited crops has prompted a renewed urgency to provide sound evidence to support science-based regulatory decisions. Interfering RNA is one of the oldest biotechnology strategies in crops available and has a long track record of successful use. In this study, we carried out a comprehensive, field-based study to assess the stability, efficacy, and risks associated with RNAi technology.

### Stable Resistance and sRNA Populations in ‘HoneySweet’

‘HoneySweet’ trees subject to intense virus infection pressure *via* grafted limbs remained virus-free even after more than a decade of virus exposure (Figure 1). The sRNA profiles of both inoculated and uninoculated ‘HoneySweet’ trees were relatively constant even when the graft inoculum was removed (Supplementary Data Sheet 1: Individual Tree sRNAs Graft Challenged). While the levels of CP derived sRNA from ‘HoneySweet’ were only slightly lower than that derived from the CP of virus-infected leaf material in susceptible control trees, overall levels of viral sRNA were nearly 25-fold higher in PPV infected controls, thereby spanning the entire PPV genome (Supplementary Data Sheet 2: Individual sRNAs for Multi-location; Supplementary Table 2, Figure 3B). The composition of the sRNA derived from transgene CP and viral CP were similar with the exception of an increase in the 24-nt sRNA species in ‘HoneySweet’ and a higher proportion of 22-nt species in virus-infected controls (Supplementary Figure 3). Surprisingly, the overlapping regions of the CP ORF produced most of the sRNA in both the transgene and virus-infected conditions, although the specific sRNA species within the regions varied according to location, tissue, and sample (Supplementary Table 3). Collectively these data show that PPV infection pressure has little to no influence on the overall levels and composition of transgene-derived sRNA. And that hairpin transgene expression of CP sequence produces sRNAs that are lower in abundance than that found in virus-infected samples, but the sequences are consistent with that produced under natural infection except with respect to sRNA length – with 24-nt species being proportionally higher in ‘HoneySweet’ while 22-nt species were proportionally higher in virus-infected ‘Stanley’ trees.

### No Long-Term Effect on Virus Populations

It is currently unknown if viruses exposed to RNAi-protected plants are subject to adaptive evolutionary pressures that could potentially break down virus resistance over time (Tepfer et al., 2015). To assess this possibility, virus-infected limbs grafted to ‘HoneySweet’ trees for over 10 years were analyzed for potential influences on viral populations. The viral CP, that is the target of ‘HoneySweet’ transgene-derived sRNA, displayed relatively few nucleotide changes (2-6), suggesting that there was little to no accumulation of variants that could potentially evade sRNA targeting by ‘HoneySweet’ (Figure 3, Supplementary Data Sheet 4: Base changes in PPV-REC viral genome). There were, likewise, no consistent impacts on the sequences of viral ORFs known to play a role in silencing suppression including HCPro and CI (Ivanov et al., 2016; Cheng and Wang, 2017; Rodamilans et al., 2018). The results show that long-term exposure to RNAi-expressing plants does not appear to impose any clear viral selection pressure. It is important to note that these findings do not exclude the potential mechanisms associated with the proliferation of naturally occuring viral strains or isolates that could potentially evade RNAi resistance due to naturally occurring low levels of target sequence similarity. To date, this is not known to occur for PPV strains that have highly conserved CP sequences.

### Safe Consumption of ‘HoneySweet’

Potential risks associated with the consumption of sRNA have been recently raised. A handful of studies have provided evidence that sRNAs may have off-target effects when consumed (Zhang Y. et al., 2012; Liu et al., 2017). These mechanisms presumably involve the silencing of genes that have sufficient levels of homology to plant-derived sRNA sequences. Here, we evaluated the health risks associated with ‘HoneySweet’ consumption and the potential for specific off-target effects in mice. First, we characterized the sRNA profiles of both leaves and ripe fruits and compared them to leaves and fruits of susceptible infected control plums (Supplementary Table 2). The results showed that the levels of viral CP-specific sRNA derived from transgene expression were lower than that produced by natural virus infection and were proportionally lower than many endogenous plant sRNAs. However, the overall levels of sRNA generated from PPV infection were significantly higher than ‘HoneySweet’ due to sRNA production across the entire length of the viral genome. In the ripe fruit of susceptible trees, the viral sRNA accounted for approximately 35% of all sRNA produced in the fruit (Figure 2; Supplementary Table 2). These data imply that the sRNA produced *via* the ‘HoneySweet’ transgene are proportionally lower than that produced by natural virus infection and make up a relatively low proportion of the total pool of sRNA in the plant. Next, we performed a comprehensive 90-day feeding study in mice that included physical and behavioral records, blood chemistries, and detailed pathological assessments of 38 different organs and tissues (Figure 3, Tables 1, 2; Supplementary Tables 4–7). We found no evidence of any negative health impacts associated with ‘HoneySweet’ plum consumption in male or female mice. Likewise, we found no evidence for any pathologies associated with predicted off-target effects based on the similarity between the most abundant ‘HoneySweet’ sRNA species and the mouse genome (Table 3). Collectively, the data did not reveal any evidence of specific health or environmental risks and demonstrated the long-term efficacy of RNAi strategies to protect plants against virus infection.

## Data Availability Statement

The datasets presented in this study can be found in online repositories. The names of the repository/repositories and accession number(s) can be found below: https://www.ncbi.nlm.nih.gov/genbank/PRJNA741990; https://www.ncbi.nlm.nih.gov/genbank/PRJNA741796; https://www.ncbi.nlm.nih.gov/genbank/PRJNA742049.

## Ethics Statement

The animal study was reviewed and approved by Institutional Animal Care and Use Committee at Oklahoma State University.

## Author Contributions

AMC, BJS, RS, JP, JKK, and CD conceived and designed the project. KS, AMC, BJS, TM, JJ, and EB performed the experiments. KS, AMC, BJS, TM, RS, JJ, EB, and CD analyzed the data. JK and CD directed aspects of the project. KS, AMC, BJS, RS, JKK, and CD wrote the manuscript. All authors reviewed the manuscript.

## Funding

The United States Department of Agriculture-Agriculture Research Service funded this project through in-house funding, work from the JKK lab was supported by the Czech Republic Ministry of Education, Youth and Sports LH15105 grant, to JKK and the reagents and sequencing were supported by grants from the United States Department of Agriculture-Foreign Agricultural Service TASC to CD, RS, and AMC.

## Conflict of Interest

The authors declare that the research was conducted in the absence of any commercial or financial relationships that could be construed as a potential conflict of interest.

## Publisher's Note

All claims expressed in this article are solely those of the authors and do not necessarily represent those of their affiliated organizations, or those of the publisher, the editors and the reviewers. Any product that may be evaluated in this article, or claim that may be made by its manufacturer, is not guaranteed or endorsed by the publisher.

## References

[B1] Beachy R. N. (1997). Mechanisms and applications of pathogen-derived resistance in transgenic plants. Curr. Opin. Biotech. 8, 215–220. 10.1016/S0958-1669(97)80105-X 9079736

[B2] Chan S. Y. Snow J. W. (2017). Formidable challenges to the notion of biologically important roles for dietary small RNAs in ingesting mammals. Genes Nutr. 12:13. 10.1186/s12263-017-0561-7 29308096 PMC5753850

[B3] Cheng X. Wang A. (2017). The potyvirus silencing suppressor protein VPg mediates degradation of SGS3 via ubiquitination and autophagy pathways. J. Virol. 91, e01478–e01416. 10.1128/JVI.01478-16 27795417 PMC5165207

[B4] Davis D. B. Doherty K. R. Delmonte A. J. McNally E. M. (2002). Calcium-sensitive phospholipid binding properties of normal and mutant ferlin C2 domains. J Biol. Chem. 277, 22883–22888. 10.1074/jbc.M201858200 11959863

[B5] Dickinson B. Zhang Y. Petrick J. S. Heck G. Ivashuta S. Marshall W. S. (2013). Lack of detectable oral bioavailability of plant microRNAs after feeding in mice *Nat*. Biotechnol. 31:965. 10.1038/nbt.2737 24213763

[B6] Fire A. SiQun X. Montgomery M. K. Kostas S. A. Driver S. E. Mello C. C. (1998). Potent and specific genetic interference by double-stranded RNA in *Caenorhabditis elegans*. Nature 391:806. 10.1038/35888 9486653

[B7] García J. A. Glasa M. Cambra M. Candresse T. (2014). Plum pox virus and sharka: a model potyvirus and a major disease. Mol. Plant Pathol. 15, 226–241. 10.1111/mpp.12083 24102673 PMC6638681

[B8] Gonsalves D. (1998). Control of papaya ringspot virus in papaya: a case study. Annu. Rev. Phytopathol. 36, 415–437. 10.1146/annurev.phyto.36.1.415 15012507

[B9] Gottula J. Fuchs M. (2009). Toward a quarter century of pathogen-derived resistance and practical approaches to plant virus disease control. Adv. Virus Res. 75, 161–183. 10.1016/S0065-3527(09)07505-8 20109666

[B10] Halloran B. P. Wronski T. J. VonHerzen D. C. Chu V. Xia X. Pingel J. E.. (2010). Dietary dried plum increases bone mass in adult and aged male mice. J. Nutr. 140, 1781–1787. 10.3945/jn.110.124198 20739449

[B11] Hamilton A. J. Baulcombe D. C. (1999). A species of small antisense RNA in posttranscriptional gene silencing in plants. Science 286, 950–952. 10.1126/science.286.5441.950 10542148

[B12] Hartmann W. Neumüller M. (2006). Breeding for resistance: breeding for Plum pox virus resistant plums (*Prunus domestica* L.) in Germany. Bull. OEPP 36, 332–336. 10.1111/j.1365-2338.2006.01010.x

[B13] He X. Semenov M. Tamai K. Zeng X. (2004). LDL receptor-related proteins 5 and 6 in Wnt/β-catenin signaling: arrows point the way. Development 131, 1663–1677. 10.1242/dev.01117 15084453

[B14] Heinemann J. A. Agapito-Tenfen S. Z. Carman J. A. (2013). A comparative evaluation of the regulation of GM crops or products containing dsRNA and suggested improvements to risk assessments. Environ. Int. 55, 43–55. 10.1016/j.envint.2013.02.010 23523853

[B15] Ivanov K. I. Eskelin K. Bašić M. De S. Lõhmus A. Varjosalo M.. (2016). Molecular insights into the function of the viral RNA silencing suppressor HCP ro. Plant J. 85, 30–45. 10.1111/tpj.13088 26611351

[B16] Jarošová J. Gadiou S. Polák J. Ravelonandro M. Scorza R. Kumar J. K. (2010). Evaluation of transgenic *Prunus domestica* L., clone C5 resistance to Plum pox virus. Julius-Kühn-Archiv 427:330.

[B17] Leonard S. P. Powell J. E. Perutka J. Geng P. Heckmann L. C. Horak R. D.. (2020). Engineered symbionts activate honey bee immunity and limit pathogens. Science 367, 573–576. 10.1126/science.aax9039 32001655 PMC7556694

[B18] Liu C. C. Prior J. Piwnica-Worms D. Bu G.. (2010). LRP6 overexpression defines a class of breast cancer subtype and is a target for therapy. Proc. Natl. Acad. Sci. USA. 107, 5136–5141. 10.1073/pnas.0911220107 20194742 PMC2841938

[B19] Liu C. C. Tsai C. W. Deak F. Rogers J. Penuliar M. Sung Y. M.. (2014). Deficiency in LRP6-mediated Wnt signaling contributes to synaptic abnormalities and amyloid pathology in Alzheimer's disease. Neuron 84, 63–77. 10.1016/j.neuron.2014.08.048 25242217 PMC4199382

[B20] Liu Y.-C. Chen W. L. Kung W.-H. Huang H.-D. (2017). Plant miRNAs found in human circulating system provide evidences of cross kingdom RNAi. BMC Genomics 18, 112. 10.1186/s12864-017-3502-3 28361700 PMC5374554

[B21] Mat Jalaluddin N. S. Othman R. Y. Harikrishna J. A. (2018). Global trends in research and commercialization of exogenous and endogenous RNAi technologies for crops. Crit. Rev. Biotechnol. 39, 1–12. 10.1080/07388551.2018.1496064 30198341

[B22] Petrick J. S. Moore W. M. Heydens W. F. Koch M. S. Sherman J. H. Lemke S. L. (2015). A 28-day oral toxicity evaluation of small interfering RNAs and a long double-stranded RNA targeting vacuolar ATPase in mice. Regul. Toxicol. Pharmacol. 71, 8–23. 10.1016/j.yrtph.2014.10.016 25445299

[B23] Polák J. Kundu J. K. Krška B. Beoni E. Komínek P. Pívalová J.. (2017). Transgenic plum *Prunus domestica* L., clone C5 (cv. HoneySweet) for protection against sharka disease. J. Integr. Agric. 16, 516–522. 10.1016/S2095-3119(16)61491-0

[B24] Rashdan N. A. Rutsch F. Kempf H. Váradi A. Lefthériotis G. MacRae V. E. (2016). New perspectives on rare connective tissue calcifying diseases. Curr. Opin. Pharmacol. 28, 14–23. 10.1016/j.coph.2016.02.002 26930168

[B25] Rimbaud L. Dallot S. Gottwald T. Decroocq V. Jacquot E. Soubeyrand S.. (2015). Sharka epidemiology and worldwide management strategies: learning lessons to optimize disease control in perennial plants. Annu. Rev. Phytopathol. 53, 357–378. 10.1146/annurev-phyto-080614-120140 26047559

[B26] Rodamilans B. Valli A. Mingot A. San León D. López-Moya J. J. García J. A. (2018). An atypical RNA silencing suppression strategy provides a snapshot of the evolution of sweet potato-infecting potyviruses. Sci. Rep. 8, 1–10. 10.1038/s41598-018-34358-y 30374036 PMC6206096

[B27] Ruiz M. T. Voinnet O. Baulcombe D. C. (1998). Initiation and maintenance of virus-induced gene silencing. Plant Cell 10, 937–946. 10.1105/tpc.10.6.937 9634582 PMC144041

[B28] Scorza R. Callahan A. Levy L. Damsteegt V. Webb K. Ravelonandro M. (2001). Post-transcriptional gene silencing in plum pox virus resistant transgenic European plum containing the Plum pox potyvirus coat protein gene. Trans. Res. 10, 201–209. 10.1023/A:1016644823203 11437277

[B29] Scorza R. Ravelonandro M. Callahan A. Zagrai I. Polak J. Malinowski T.. (2016). ‘HoneySweet’(C5), the first genetically engineered plum pox virus–resistant Plum (*Prunus domestica* L.) Cultivar. HortScience 51, 601–603. 10.21273/HORTSCI.51.5.601

[B30] Scorza R. Ravelonandro M. Callahan A. M. Cordts J. M. Fuchs M. Dunez J.. (1994). Transgenic plums (*Prunus domestica* L.) express the plum pox virus coat protein gene. Plant Cell Rep. 14, 18–22. 10.1007/BF00233291 24194220

[B31] Singh K. Kundu J. K. (2017), Variation in coat protein sequence of Wheat streak mosaic virus among crop no crop hosts. Crop Pasture Sci. 68, 328–336. 10.1071/CP17025

[B32] Singh K. Zouhar M. Mazakova J. Rysanek P. (2014), Genome Wide Identification of the Immunophilin Gene Family in Leptosphaeria maculans: a causal agent of blackleg disease in oilseed rape (Brassica napus). OMICS. 18, 645–657. 10.1089/omi.2014.0081 25259854 PMC4175974

[B33] Tepfer M. Jacquemond M. García-Arenal F. (2015). A critical evaluation of whether recombination in virus-resistant transgenic plants will lead to the emergence of novel viral diseases. New Phytol. 207, 536–541. 10.1111/nph.13358 25982848

[B34] The International Peach Genome Initiative (2013). The high-quality draft genome of peach (*Prunus persica*) identifies unique patterns of genetic diversity, domestication and genome evolution. Nat. Genet. 45, 487–494. 10.1038/ng.2586 23525075

[B35] Tosar P. Rovira C. Naya H. Cayota A. (2014). Mining of public sequencing databases supports a non-dietary origin for putative foreign miRNAs: underestimated effects of contamination in NGS. RNA 20, 754–757. 10.1261/rna.044263.114 24729469 PMC4024629

[B36] van Meurs J. B. Trikalinos T. A. Ralston S. H. Balcells S. Brandi M. L. Brixen K.. (2008). Large-scale analysis of association between LRP5 and LRP6 variants and osteoporosis. JAMA 299, 1277–1290. 10.1001/jama.299.11.1277 18349089 PMC3282142

[B37] Vilanova S. Romero C. Abbott A. G. Llacer G. Badenes M. L. (2003). An apricot (*Prunus armeniaca* L.) F2 progeny linkage map based on SSR and AFLP markers, mapping plum pox virus resistance and self-incompatibility traits. Theor Appl Genet 107, 239–247. 10.1007/s00122-003-1243-y 12845439

[B38] Zhang J. Khan S. A. Heckel D. G. Bock R. (2017). Next-generation insect-resistant plants: RNAi-mediated crop protection. Trends Biotechnol. 35, pp.871–882. 10.1016/j.tibtech.2017.04.009 28822479

[B39] Zhang L. Hou D. Chen X. Li D. Zhu L. Zhang Y.. (2012). Exogenous plant MIR168a specifically targets mammalian LDLRAP1: evidence of cross-kingdom regulation by microRNA. Cell Res. 22, 107–126. 10.1038/cr.2011.158 21931358 PMC3351925

[B40] Zhang Y. Wiggins B. E. Lawrence C. Petrick J. Ivashuta S. Heck G. (2012). Analysis of plant-derived miRNAs in animal small RNA datasets. BMC Genomics 13:381. 10.1186/1471-2164-13-381 22873950 PMC3462722

[B41] Zuriaga E. Soriano J. M. Zhebentyayeva T. Romero C. Dardick C. Cañizares J.. (2013). Genomic analysis reveals *MATH* gene(s) as candidate(s) for plum pox virus (PPV) resistance in apricot (*Prunus armeniaca* L.). Mol. Plant Path. 14, 663–677. 10.1111/mpp.12037 23672686 PMC6638718

