# Supplementary material for: Long-Term Efficacy and Safety of RNAi-Mediated Virus Resistance in ‘HoneySweet’ Plum
Source: Front Plant Sci. 2021 Oct 12;12:726881. doi: 10.3389/fpls.2021.726881 (PMC8546108; doi:10.3389/fpls.2021.726881)
Supplement: Supplementary file 6 [file Data_Sheet_6.pdf]

Supplementary Figure 1. ‘Honeysweet’ fruit

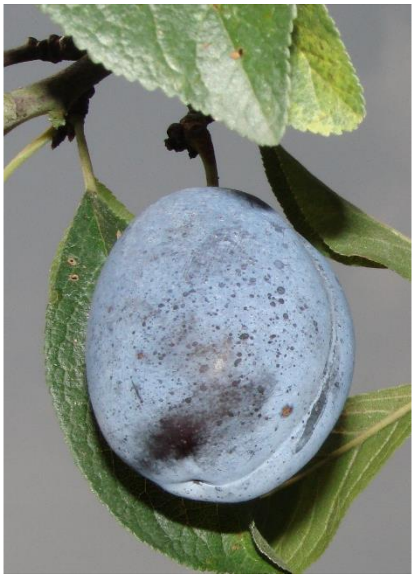

Supplementary Figure 2. PPV-REC graft inoculated 'HoneySweet' tree experiment. 'HoneySweet trees were inoculated with a graft bud of 'Emma Leppermann' infected with PPV strain REC. The graft was removed from half of the trees 9 years later. Visual symptoms of PPV infection on 'Emma Leppermann' are not easily detected, yet PCR tests were positive in the first year on while the 'HoneySweet' scion were always negative.

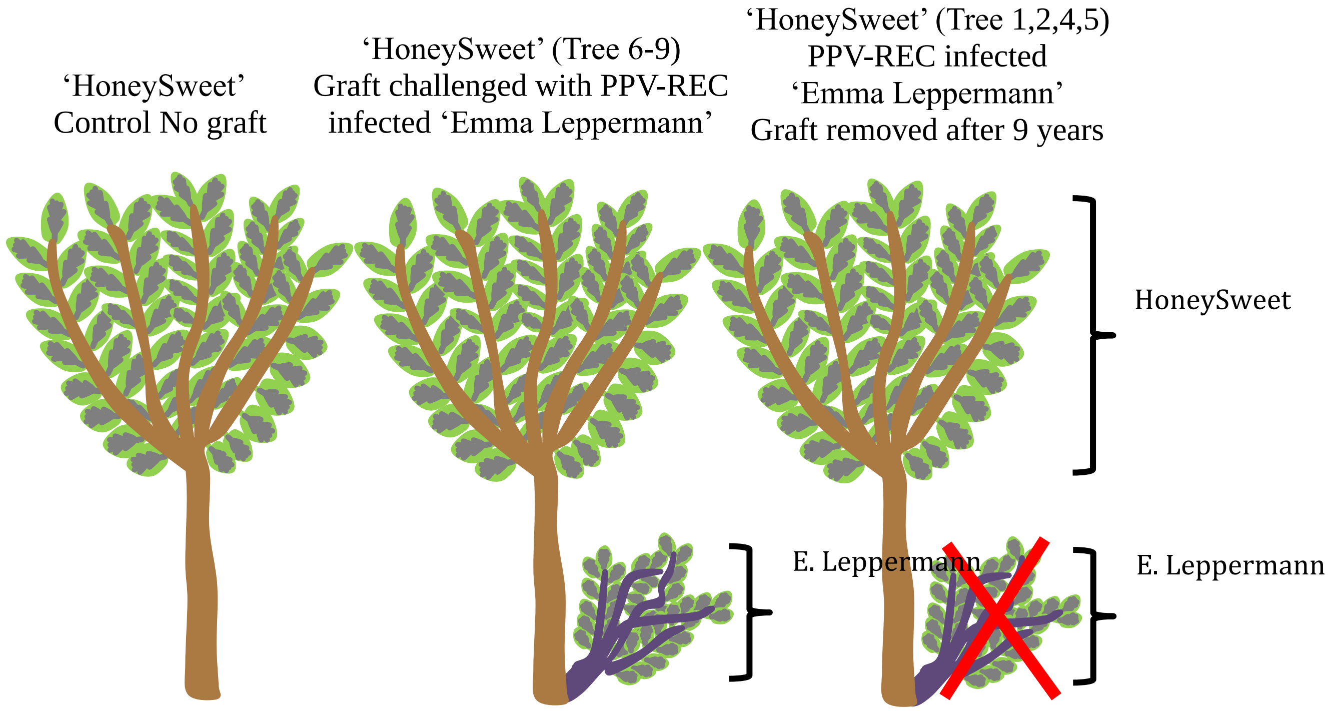


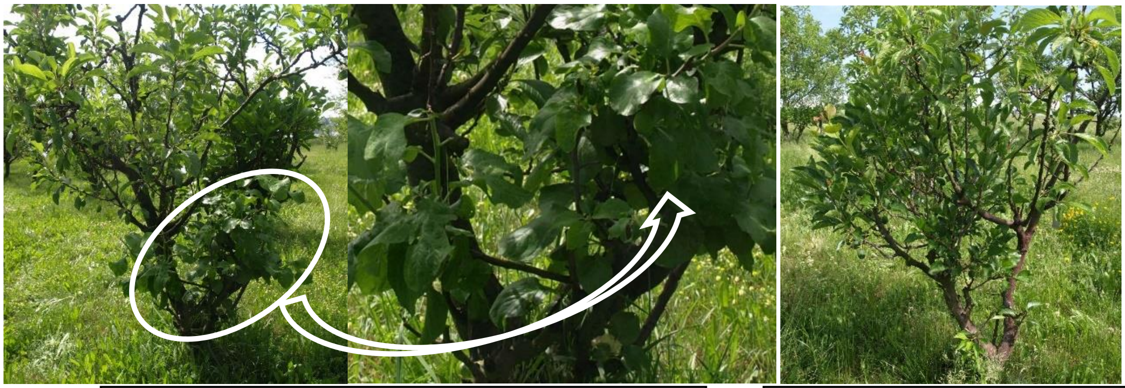

'HoneySweet' exhibiting the 'Emma Leppermann' graft in the lower part of the

'HoneySweet' tree where the graft has been removed

Supplementary Figure 3. Distribution of sRNA sizes in multi-location trees and graft infected trees.

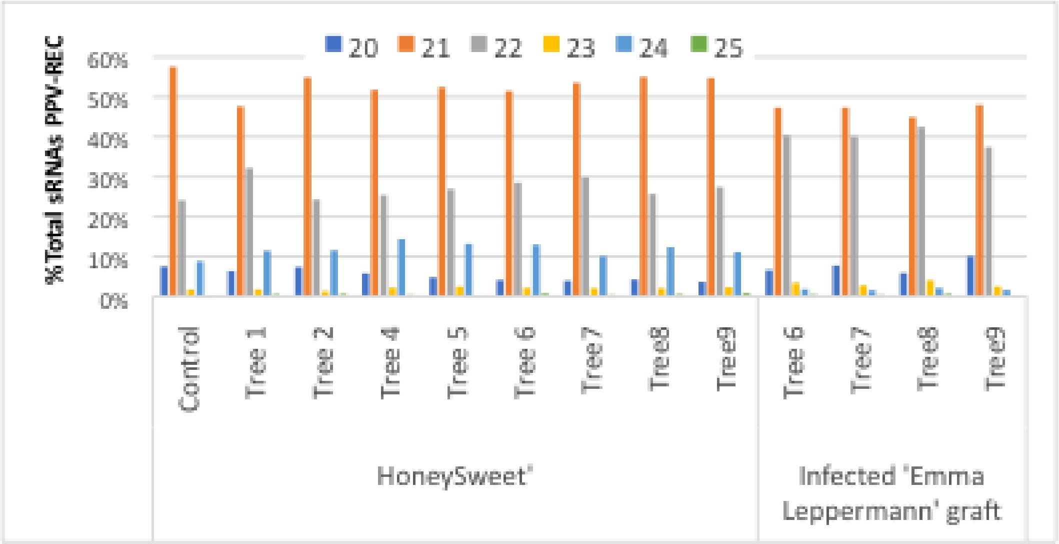


A. Size distribution in 7 individual 'HoneySweet' trees exposed to PPV-REC and control as well as 4 PPV-REC infected 'Emma Leppermann' grafts.

B. Size distribution in 'HoneySweet' and naturally infected 'Stanley' fruit and leaf from multi-locations

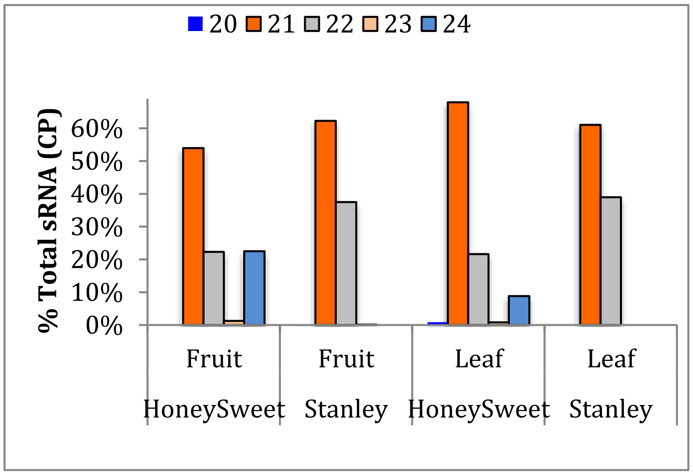


C. Size distribution in 'HoneySweet' leaf from control (full color) and graft challenged (lighter).

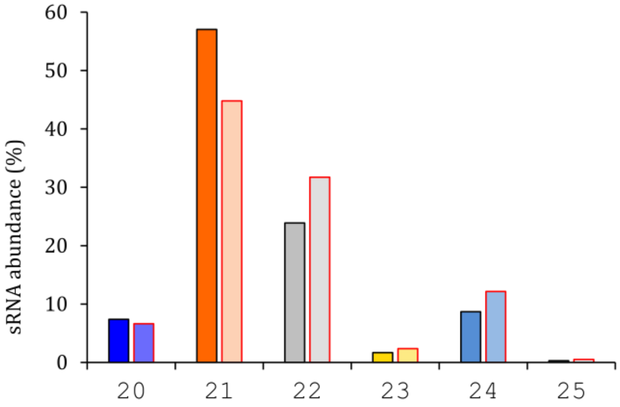

Supplementary Figure 4. PPV-CP comparison between PPV-D (D00298) and PPV-REC (JQ794501).

```
D00298       1                                           GUCCAGACGUUGUUGUGG   18
JQ794501  8701 GAUAUCUUGAAGCAUUUUACAAUGAUAUUGAUGAUAGCCUUGACUCCAACAUUGUUAUA- 8759
                                                            *  ** ***** *

D00298      19 CACCAAGCUGACGAAAGAGAGGACGAGGAGGAAGUUGAUGCAGGCAAGCCGAUUGUAGUU   78
JQ794501  8760 CACCAGGCUGAUGAAAAGGAGGACGAUGAAGAAGUCGAUGCAGGAAAACCCACUGUAGUA 8819
               ***** ***** ****  ******** ** ***** ******** ** ** * ******

D00298      79 ACUGCACCGGCAGCAACUAGCCCAAUACUUCAACCACCUCCAGUCAUACAGCCUGCACCC  138
JQ794501  8820 ACUGCACCGGCAGCAACUGUGGCAACAACUCAACCAGCUCCAGUGAUACAACCUGCAAUU 8879
               ******************    *** *  ******* ******* ***** ******

D00298     139 CGGACUACGGCGCCAAUGCUCAACCCCAUUUUCACGCCAGCAACAACUCAACCAGCAACA  198
JQ794501  8880 CAAACCACAACACCAAUGUUCAACCCCAUUUUCACUCCAGCAACAACUCAGCCUGCGAUA 8939
               *  ** **  * ****** **************** ************** ** ** * *

D00298     199 AAACCAGUUUCACAGGUGCCAGGACCUCAACUGCAAACUUUUGGAACAUAUGGUAAUGAG  258
JQ794501  8940 AGACCAGUUUCUCCAAUUUCAGGGGCCACACCGCAGUCUUUUGGAGUUUAUGGAAAUGAG 8999
               * ********* *   *  ****  *   ** ***  ********   ***** ******

D00298     259 GAUGCAUCACCUAGCAACUCAAACGCGCUAGUCAACACAAACAGAGACAGGGACGUCGAU  318
JQ794501  9000 GAUGCAUCACCUAGCACCUCAAACACUUUGGUGAACACAGGAAGGGAUAGGGACGUCGAU 9059
               **************** ******* *  * ** ******   ** ** ************

D00298     319 GCAGGAUCAAUUGGAACUUUUACAGUGCCACGUUUGAAGGCAAUGACUUCGAAACUAUCU  378
JQ794501  9060 GCAGGAUCGAUUGGAACUUUCGCAGUGCCACGCUUAAAAACAAUGACAUCGAAGUUAUCU 9119
               ******** ***********  ********** ** **  ******* *****  *****

D00298     379 CUGCCAAAGGUGAAGGGAAAGGCUAUUAUGAACUUGAACCAUUUGGCACAUUAUAGUCCU  438
JQ794501  9120 CUACCGAAGGUGAAAGGAAAAGCAAUUAUGAACUUAAAUCAUUUGGCACAGUACAGUCCU 9179
               ** ** ******** ***** ** *********** ** *********** ** ******

D00298     439 GCACAGGUUGACUUGUCAAACACGAGAGCUCCGCAGUCUUGUUUCCAAACUUGGUAUGAA  498
JQ794501  9180 GCACAAGUUGAUUUAUCAAACACACGAGCUCCACAAUCCUGUUUCCAGACUUGGUAUGAA 9239
               ***** ***** ** ********  ******* ** ** ******** ************

D00298     499 GGAGUUAAGCGAGAUUAUGAUGUCACGGACGAUGAAAUGAGCAUCAUUUUAAAUGGUCUU  558
JQ794501  9240 GGAGUUAAGCGUGAUUACGAUGUCACAGAUGAGGAAAUGAGCAUCAUUUUGAAUGGCCUG 9299
               *********** ***** ******** ** ** ***************** ***** **

D00298     559 AUGGUUUGGUGCAUAGAGAAUGGAACAUCCCCGAAUAUCAAUGGAAUGUGGGUGAUGAUG  618
JQ794501  9300 AUGGUUUGGUGCAUUGAAAACGGAACGUCUCCUAACAUCAACGGAAUGUGGGUGAUGAUG 9359
               ************** ** ** ***** ** ** ** ***** ******************

D00298     619 GAUGGGGAAACACAAGUGGAGCAUCCAAUAAAGCCAUUGUUGGAUCAUGCGAAACCCACU  678
JQ794501  9360 GAUGGGGAAACACAAGUGGAGUAUCCAAUAAAGCCAUUGUUGGAUCACGCGAAACCCACU 9419
               ********************* ************************* ************

D00298     679 UUUAGACGAAUUGUGGCACGUUUCAGCGACGUGGCCGAAGCGUGUGUGGAAAAACGAAAU  738
JQ794501  9420 UUUAGACAAAUUAUGGCACAUUUCAGUAACGUGGCUGAAGCGUAUAUUGAAAAACGGAAU 9479
               ******* **** ****** ******  ******* ******* * * ******** ***

D00298     739 UACGAAAAAGCAUACAUGCCAAGGUAUGGAAUUCAGCGCAACCUGACAGACUACAGCCUC  798
JQ794501  9480 UACGAGAAAGCAUACAUGCCAAGGUAUGGAAUUCAGCGCAACCUGACAGAUUACAGCCUC 9539
               ***** ******************************************** *********

D00298     799 GCCAGAUAUGCCUUUGAUUUUUACGAAAUGACUUCAACGACACCCGUACGGGCACGUGAA  858
JQ794501  9540 GCCAGAUACGCCUUUGAUUUUUACGAAAUGACUUCAACAACGCCUGUGCGUGCACGUGAA 9599
               ******** ***************************** ** ** ** ** *********

D00298     859 GCUCAUAUCCAGAUGAAGGCAGCAGCAUUGAGAAAUGUUCAAAAUCGUUUAUUUGGCUUG  918
JQ794501  9600 GCUCAUAUACAGAUGAAGGCAGCAGCAUUGAGAAAUGUUCAGAAUCGUUUAUUUGGCUUG 9659
               ******** ******************************** ******************
```

```
D00298     919 GAUGGAAACGUCGGAACACAGAAGCAGGACACAGAGAGACACACGGAUGGUGAUGUUAAU  978
JQ794501  9660 GAUGGAAACGUCGGAACACAAGAAGAGGACACAGAGAGACACACCGCUGGUGACGUGAAU 9719
               ********************  *  ******************* * ****** ** ***

D00298     979 CGCAACAUGCACACCUUCCUCGGUGUGAGGGGAGUGUAGUGGUCUCGGUAUCUAUCAUAA 1038
JQ794501  9720 CGCAACAUGCACAACCUCCUCGGUGUGAGGGGAGUGUAGUGGUCUCGGUAUCUAUCAUAA 9779
               ************* * ********************************************

D00298    1039 ACUCUACCUGG-UGAGAGUCUAAUCAUCCAGUUG-UUUUAGAUUCCUGUUAGCAUCCUUU 1096
JQ794501  9780 ACUCUACUUGGGUGAGAGUCUAGUCACCCAAUUGGUUUUAGAUUCCUGUUAGCAUCCUUU 9839
               ******* *** ********** *** *** *** *************************

D00298    1097 UCUCCGCUUUAAUAGCAGUACAUUCAGUGAGGUUUUACCUCCAUAUGUUCUAGUCUGUUA 1156
JQ794501  9840 UCUCCGCUUUAAUUGCGGCACAUUCAGUGAGGUUGUACCUCCACAUGUGUUAGUCUUUUA 9899
               ************* ** * *************** ******** ****  ****** ***

D00298    1157 UUGUCGAACACAGGCCCUUGUAUCUGAUGUAGCGAGUGCUCCACUUCAUUCGGGUUAUAG 1216
JQ794501  9900 UUGUCGAACACAGGCCCUUGUAUCUGAUGUAGCGAGUGUUUCACUCCAUUCGGGUUAUAG 9959
               ************************************** * **** **************

D00298    1217 UUCUUGUGCAAGAGAC 1232
JQ794501  9960 UUCUUGUGCAAGAGAC 9975
               ****************
```
