# Supplementary material for: Long-Term Efficacy and Safety of RNAi-Mediated Virus Resistance in ‘HoneySweet’ Plum
Source: Front Plant Sci. 2021 Oct 12;12:726881. doi: 10.3389/fpls.2021.726881 (PMC8546108; doi:10.3389/fpls.2021.726881)
Supplement: Supplementary file 7 [file Data_Sheet_7.docx]

| Supplementary Table 1. Location and number of trees used for the multi-location study | | | | | |
| --- | --- | --- | --- | --- | --- |
| Cultivar | Valencia, Spain | Praha, Czech Republic | Zaragoza, Spain | Plovdiv, Bulgaria | Kearneysville, WV, USA |
| ‘HoneySweet’ | 2 trees^1^ | 3 trees^2^ |  |  | 1 tree (3 years)^3^ |
| ‘Stanley’ | 2 trees |  | 3 trees | 2 trees | 1 tree (3 years)^3^ |
| ^1^Permissions for field release of GMO Nos. B/ES/96/16 and B/ES/05/14 (‘HoneySweet’) were given by the Spanish Ministerio de Medio Ambiente | | | | | |
| ^2^HoneySweet’ was grown under the Ministry of Environment of the Czech Republic GM planting No. 881/OER/GMO/01 and the field trial was extended, Ministry Reference Number 41538/ENV/09 issued on September 18, 2009. | | | | | |
| ^3^The same tree was sample for three years and each sample was analyzed | | | | | |

Supplementary Table 2. Levels of PPV RNA detected in 'Stanley' and 'HoneySweet' trees from multi-locations.

|  | | | | | | |
| --- | --- | --- | --- | --- | --- | --- |
| Matching Sequence | Leaf | | | Fruit | | |
| RNAseq^1^ |  |  |  |  |  |  |
|  | Stanley^2^ | Infected Stanley^3^ | HoneySweet^4^ | Stanley | Infected Stanley | HoneySweet |
| PPV^5^ | 0%^6^ | 0.778% | 0.001% | 0.000% | 2.337% | 0.001% |
| CP^7^ | 0.000% | 0.240% | 0.002% | 0.000% | 0.567% | 0.002% |
| UidA^8^ | 0.000% | 0.000% | 0.065% | 0.000% | 0.000% | 0.023% |
| NPTII^9^ | 0.000% | 0.000% | 0.002% | 0.000% | 0.000% | 0.000% |
| pBR322^10^ | 0.000% | 0.000% | 0.000% | 0.000% | 0.000% | 0.000% |
| sRNAseq^11^ |  |  |  |  |  |  |
| PPV | 0.007% | 3.645% | 2.173% | 0.001% | 35.207% | 0.306% |
| CP | 0.001% | 0.156% | 2.180% | 0.000% | 2.040% | 0.306% |
| UidA | 0.000% | 0.000% | 0.492% | 0.000% | 0.000% | 0.089% |
| NPTII | 0.000% | 0.000% | 0.022% | 0.000% | 0.000% | 0.004% |
| pBR322 | 0.000% | 0.000% | 0.138% | 0.000% | 0.000% | 0.035% |
| ^1^reads derived from sequencing polyA containing RNAs, presumably mRNAs. ^2^6 trees. ^3^4 trees. ^4^8 trees. | | | | | | |
| ^5^match any of the genes from the whole genome of PPV. ^6^% reads that map to transgenes and PPV | | | | | | |
| sequences relative to those that map to peach genome after filtering ribosomal, chloroplast and | | | | | | |
| mitochodrial RNAs out. ^7^coat protein gene only (D + M). ^8^UidA transgene. ^9^NPTII transgene. ^10^part of the | | | | | | |
| transgene vector that matches pBR322. ^11^reads derived from sequencing small RNAs >50nt | | | | | | |

Supplementary Table 3. Individual sRNAs flanking a hot spot vary in length and abundance amongst the different tissues and cultivars.


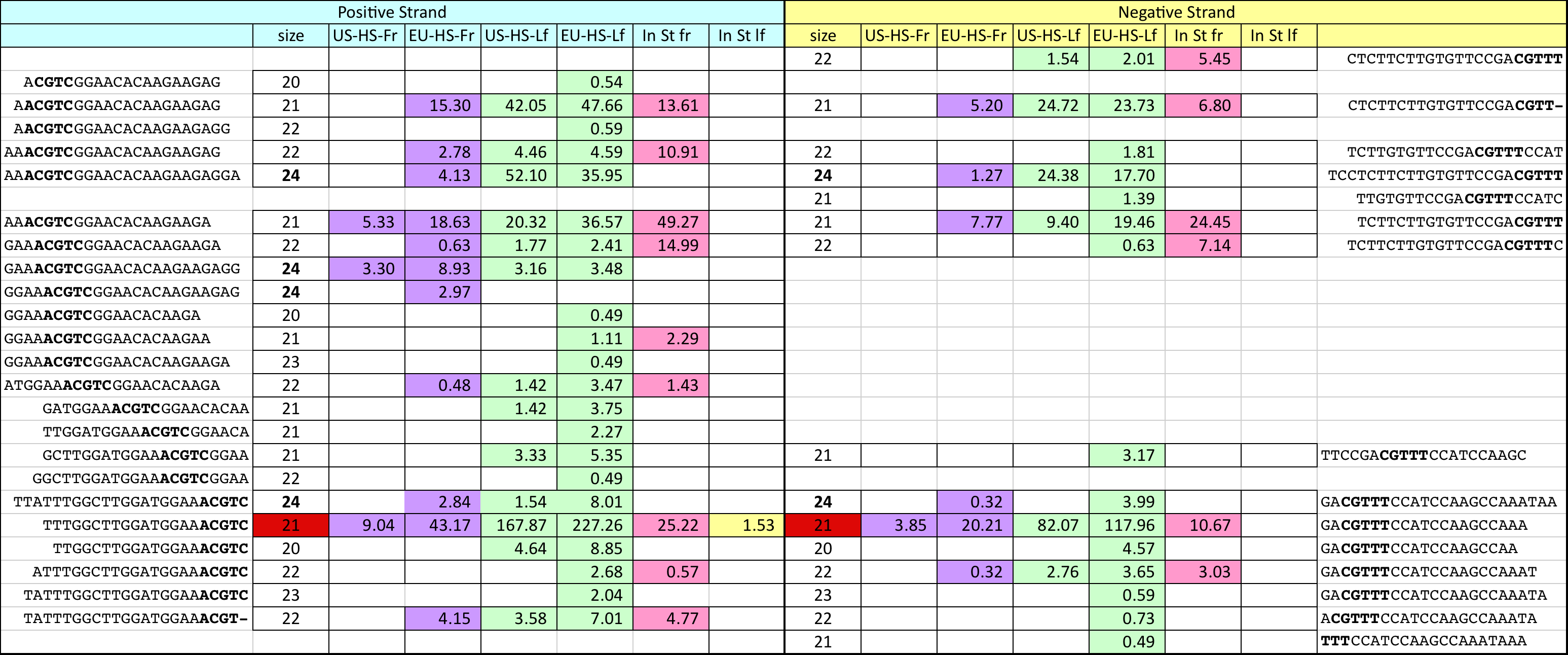


HS is ‘HoneySweet’, St is ‘Stanley’, US is the United States (No PPV), EU is the European Union, Fr is fruit tissue and Lf is leaf tissue. Red text represents sRNA found in both St and HS. Black tex are sRNAs found only in HS. Lavender shading—‘HoneySweet’ fruit. Green shading—‘HoneySweet’ leaf. Pink shading—‘Stanley’ fruit. Yellow shading—‘’Stanley’ leaf. Red Shading is the most abundant sRNA in this hot spot.

Supplementary Table 4. ‘Honeysweet’ Study Blood Chemistry for Females.

CHOL=cholesterol; TRIG=triglycerides; ALT=alanine aminotransferase; AST=aspartate aminotransferase; ALP=alkaline phosphatase; GLU=glucose; PHOS=phosphate; Ca=calcium; TBIL=total bilirubin; TP=total protein; ALB=albumin; GLOB=globins; AG Ratio=albumin/globin; BUN=blood urea nitrogen; CREAT=creatinine; Na=sodium; K=potassium; Cl=chloride; Na/K ratio=sodium/potassium

Data are means + SE; Glucose values do not represent normal fasting glucose due to anesthesia;

-- indicates no normal range for the mouse.

Mouse #116 in DP-15% ALT very high values; Results shown with and without #116

Supplementary Table 5. Total White Blood Cells and White Blood Cell Differentials for Male Mice.

Supplementary Table 6. Total White Blood Cells and White Blood Cell Differentials for Female Mice.

Supplementary Table 7. Pathology Report Female Mice.
